# Gut Bacterium *Lysinibacillus Sphaericus* Exacerbates Aspirin‐induced Intestinal Injury by Production of Carboxylesterase EstB

**DOI:** 10.1002/advs.202517747

**Published:** 2025-12-12

**Authors:** Zeyu Zhao, Qing Li, Xiaowu Bai, Ertao Zhai, Weigang Dai, Yan Qian, Tianhao Zhang, Zhixin Huang, Ziyu Huang, Fangang Meng, Jianhui Chen, Tao Zuo, Shirong Cai, Risheng Zhao

**Affiliations:** ^1^ Division of Gastrointestinal Surgery Center the First Affiliated Hospital Sun Yat‐sen University Guangzhou Guangdong 510080 China; ^2^ Ministry of Education Key Laboratory of Human Microbiome and Chronic Diseases (Sun Yat‐sen University) Guangzhou Guangdong 510655 China; ^3^ Dermatology hospital of Southern Medical University Guangzhou Guangdong 510091 China; ^4^ Department of General Surgery Nanfang Hospital Southern Medical University Guangzhou Guangdong 510515 China; ^5^ Guangdong Institute of Gastroenterology the Sixth Affiliated Hospital Sun Yat‐sen University Guangzhou Guangdong 510655 China; ^6^ Orthopedics sports medicine the First Affiliated Hospital Sun Yat‐sen University Guangzhou Guangdong 510080 China

**Keywords:** aspirin, drug metabolism, intestinal barrier function, microbiome

## Abstract

Aspirin provides long‐term health benefits but can cause gastrointestinal toxicity, and the role of gut microbiota in aspirin metabolism and enterotoxicity remains unclear. In this study, the contribution and mechanisms of microbiota–aspirin interactions in intestinal injury are investigated. In a mouse model, aspirin‐induced enteropathy is found to be more severe in microbiota‐replete than in microbiota‐depleted mice, implicating a detrimental role of gut microbiota. Co‐cultivation experiments revealed that gut microbes facilitated the biotransformation of aspirin into salicylic acid, a metabolite more harmful than aspirin itself in disrupting epithelial cell integrity and renewal, both in vitro and in vivo. Through metagenomic screening, selective bacterial interrogation, and functional validation, *Lysinibacillus sphaericus* is identified as the culprit bacterium, and its secreted carboxylesterase EstB as the key enzyme catalyzing aspirin hydrolysis to salicylic acid. Importantly, inhibition of microbial EstB with the dietary compound flavanomarein abrogated aspirin biotransformation and prevented intestinal injury. Together, these findings reveal *L. sphaericus* and EstB as central drivers of aspirin enterotoxicity, highlight the functional importance of gut microbiota in drug metabolism, and suggest microbiota‐ and metabolite‐guided precision prevention strategies.

## Graphical abstract

Schematic overview illustrating the detrimental role of gut microbiota in aspirin‐induced intestinal injury. *L. sphaericus* and its secreted carboxylesterase EstB are identified as key drivers that catalyze aspirin hydrolysis into salicylic acid, thereby exacerbating intestinal injury. Inhibition of EstB by the dietary compound flavanomarein effectively blocked aspirin biotransformation and alleviated intestinal toxicity, highlighting a potential microbiota‐targeted strategy for prevention.

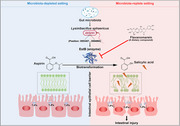

## 1 Introduction

Aspirin, a quintessential nonsteroidal anti‐inflammatory drug (NSAID), provides significant health benefits due to its multifaceted therapeutic effects. However, these advantages are often overshadowed by adverse effects associated with long‐term use. The most common and clinically significant complication is gastrointestinal toxicity, which manifests as mucosal erosion or ulceration and can progress to severe bleeding or perforation.^[^ ^1^, ^2^ ^]^ In severe cases, these complications may compel patients to discontinue therapy, depriving them of aspirin's beneficial effects.^[^ ^3^ ^]^ Endoscopic studies reveal that 11% of chronic aspirin users have gastric or duodenal ulcers, with 80% of these individuals remaining asymptomatic.^[^ ^4^ ^]^ Furthermore, capsule endoscopy demonstrates intestinal injury in 88.5% to 100% of patients, with erosions or ulcers observed in 68%.^[^ ^5^ ^]^ Although proton pump inhibitors (PPIs) effectively reduce aspirin‐induced gastropathy and are widely used as adjunct therapy,^[^ ^6^, ^7^ ^]^ paradoxically, they can worsen aspirin‐associated enterotoxicity.^[^ ^8^, ^9^ ^]^ Current therapeutic options remain limited, with misoprostol showing modest efficacy in treating aspirin‐related enteropathy.^[^ ^10^ ^]^ Understanding the underlying mechanisms is crucial, as this knowledge could pave the way for more effective interventions.

When administered orally, aspirin first encounters the complex and dynamic environment of the gastrointestinal (GI) tract before reaching systemic circulation. This microenvironment hosts a vast and diverse microbiota, which has been shown to influence drug toxicity across multiple organs.^[^ ^11^ ^]^ Studies on rodents treated with NSAIDs reveal that these drugs induce intestinal ulcers in conventional rats, but not in germ‐free or antibiotic‐treated animals, suggesting a microbiota‐dependent mechanism.^[^ ^12^, ^13^ ^]^ Moreover, colonization with probiotic bacteria such as *Bifidobacterium adolescentis* or *Lactobacillus acidophilus* has been shown to reduce NSAIDs‐related intestinal injury, whereas colonization with *Eubacterium limosum* or *Escherichia coli* exacerbates it.^[^ ^13^ ^]^ Further, remodeling gut microbiota with antibiotics like ampicillin can markedly reduce NSAIDs‐induced enterotoxicity,^[^ ^14^ ^]^ strengthening the evidence for a microbiota‐driven process. While these findings suggest that NSAIDs‐induced intestinal injury may involve the gut microbiota, it remains unclear whether the same holds true for aspirin, a simple and widely used NSAID. It is especially important to further explore whether interactions between gut microbiota and aspirin influence the drug's toxicity profile.

Recent advances reveal that gut bacteria produce a diverse array of bioactive enzymes that mediate drug biotransformation, a key process shaping drug efficacy and toxicity.^[^ ^15^ ^]^ Notably, bacterial β‐glucuronidases contribute to irinotecan‐induced diarrhea by reactivating toxic metabolites through glucuronide removal, and inhibition of these enzymes mitigates the resulting enterotoxicity.^[^ ^16^ ^]^ Similarly, inhibition of this bacterial enzyme by amoxapine reduces the hydrolysis of mycophenolic acid's glucuronide metabolite, decreasing associated enteropathy.^[^ ^17^ ^]^ These examples underscore the importance of microbial biotransformation in drug‐induced intestinal injury. Our findings, together with those of others, suggest that aspirin undergoes microbial biotransformation: depletion of the gut microbiota increases the bioavailability of oral aspirin, whereas this effect is not observed with intravenous administration, which bypasses microbial interactions.^[^ ^18^, ^19^ ^]^ Whether microbiota‐mediated biotransformation enhances aspirin's intestinal toxicity warrants further study. Here, we investigate the role of gut microbiota in aspirin‐induced intestinal injury and show how microbiota–aspirin interactions drive enterotoxicity, providing new insights into microbial influences on aspirin toxicity and potential strategies for its prevention.

## 2 Results

### 2.1 Gut Microbiota Exacerbate Aspirin‐Induced Intestinal Injury

**Figure 1:**
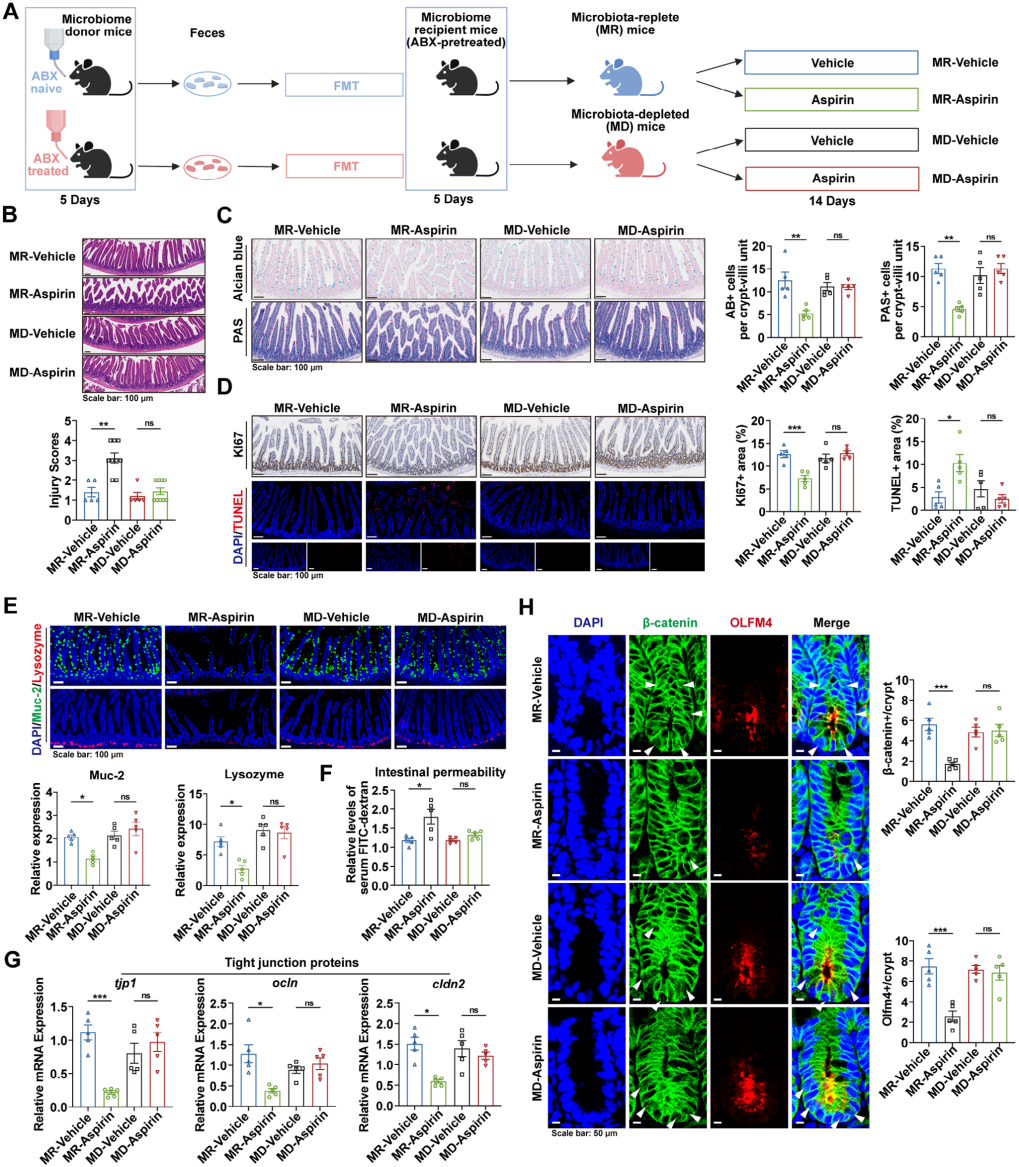
Gut microbiota exacerbates aspirin‐induced intestinal injury. A) Schematic of MR and MD mouse establishment. Mice were gavaged with vehicle or aspirin before assessment. Created with BioRender.com. B) Representative H&E images and quantified injury scores (n = 5–9). C) Alcian blue and PAS images with quantification of AB+ and PAS+ cells per crypt‐villus (n = 5). D) Ki67 and TUNEL staining images, with quantitative analysis of proliferative and apoptotic areas (n = 5). E) Immunofluorescence staining of Muc2 (green) and lysozyme (red) with quantification of positive cells (n = 5). F) Serum FITC‐dextran to assess permeability (n = 5). G) Expression of *tjp1*, *ocln*, and *cldn2* in the small intestine (n = 5). H) Immunofluorescence images of β‐catenin (nuclear β‐catenin indicated by arrows) and Olfm4, with quantification of positive cells per intestinal crypt (n = 5). *p* values were calculated using one‐way ANOVA or Kruskal–Wallis test. ^*^ *p* < 0.05; ^**^ *p* < 0.01; ^***^ *p* < 0.001. MR: microbiota replete; MD: microbiota depleted; ABX: antibiotics; FMT: fecal microbiota transplantation.

To investigate whether gut microbiota are involved in aspirin‐induced intestinal injury, specific pathogen‐free (SPF) mice, pretreated with antibiotics, were used to generate microbiota‐replete (MR) and microbiota‐depleted (MD) groups. The MR and MD mice were established by transplanting fecal microbiota from antibiotic‐naive or antibiotic‐treated donors, respectively (**Figure** **1**A). This approach minimized potential confounding effects of antibiotics on the intestinal epithelium, ensuring that the observed outcomes primarily reflected the impact of gut microbiota. Fecal microbial DNA concentration and 16S rRNA analysis verified successful microbiota depletion by antibiotics (Figure S1A,B, Supporting Information), and 16S rRNA sequencing further confirmed a distinct microbial composition between MR and MD mice (Figure S1C–E, Supporting Information). Aspirin or vehicle was subsequently administered to MR and MD mice to evaluate its effect on intestinal injury under distinct microbiota conditions, revealing that aspirin markedly increased histological intestinal injury in MR mice, whereas no significant effect was observed in MD mice, as shown by H&E and Alcian Blue–Periodic Acid–Schiff (AB‐PAS) staining (Figure 1A–C). Consistently, aspirin significantly reduced intestinal epithelial cell viability in MR mice, but not in MD mice, as indicated by Ki67 and TUNEL staining (Figure 1D). Furthermore, in the MR setting, aspirin‐treated mice exhibited impaired barrier function, evidenced by decreased Mucin2 and lysozyme levels (Figure 1E), increased permeability reflected by circulating Fluorescein Isothiocyanate‐dextran (FITC‐dextran) levels (Figure 1F), compromised integrity demonstrated by lower expression of tight junction proteins (TJPs) (Figure 1G), and diminished regenerative capacity, as indicated by decreased β‐catenin activity and Olfm4 levels in crypt cells (Figure 1H). Despite the markedly reduced load and diversity of gut microbiota transplanted into MD mice compared to MR mice (Figure S1C–E, Supporting Information), no significant differences were found between MD and MR mice without aspirin treatment (Figure 1B–H). Collectively, these findings indicate that aspirin induces intestinal injury in a microbiota‐dependent manner, with neither aspirin nor microbiota alone causing significant damage, highlighting the role of microbiota–aspirin interactions.

### 2.2 Gut Microbiota‐Mediated Aspirin Biotransformation Contributes to Intestinal Injury

**Figure 2:**
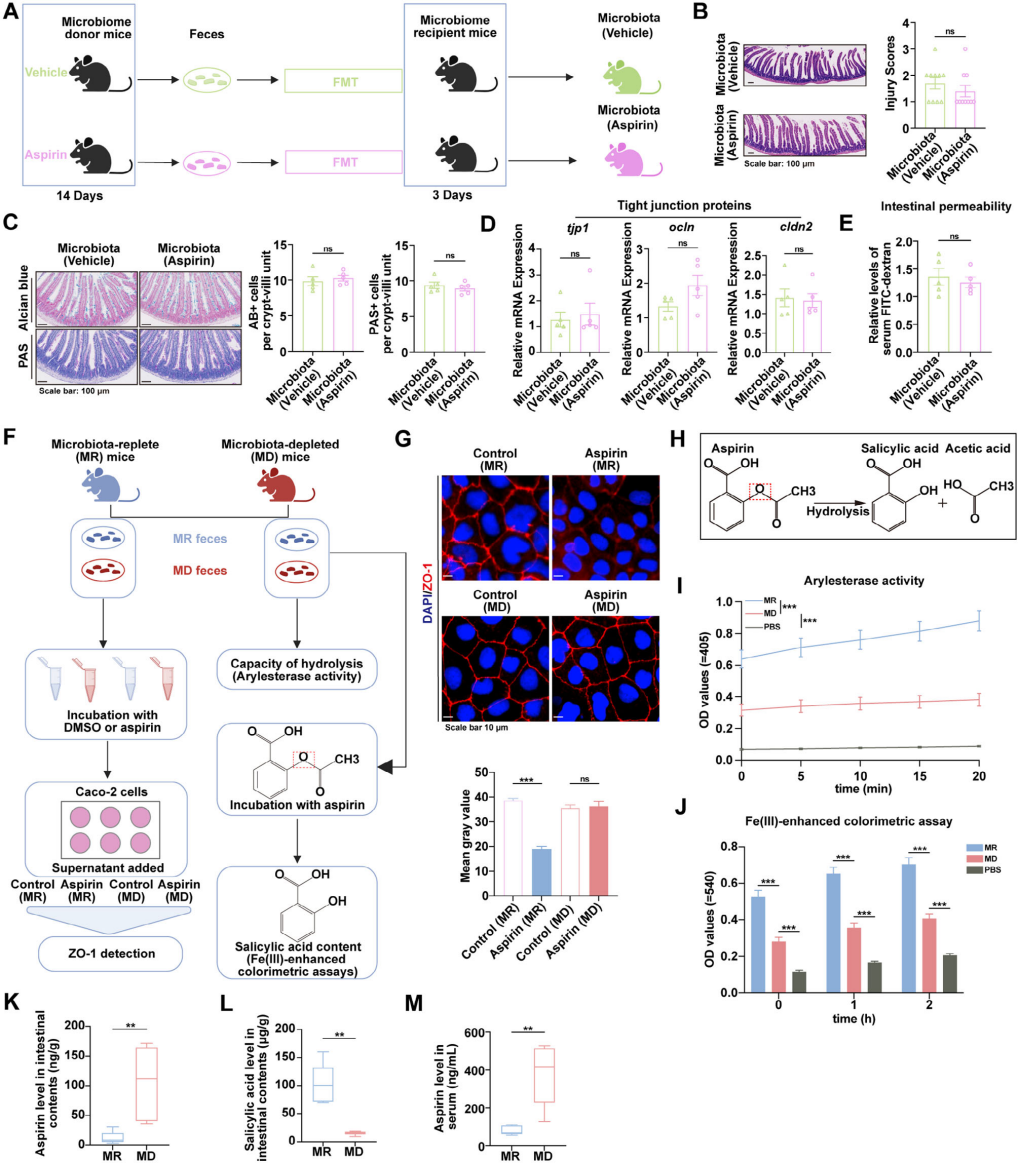
Gut microbiota‐mediated aspirin biotransformation contributes to intestinal injury. A) Schematic of the impact of aspirin‐altered gut microbiota on intestinal injury. Created with BioRender.com. B) H&E images and injury scores (n = 10). C) Alcian blue and PAS images (n = 5). D) Expression of *tjp1*, *ocln*, and *cldn2* in the small intestine (n = 5). E) Serum levels of FITC‐dextran (n = 5). F) Schematic of in vitro analysis of microbiota‐driven aspirin effects and biotransformation. Created with BioRender.com. G) Immunofluorescence analysis of ZO‐1 expression in Caco‐2 cells (n = 3). H) Illustration of the chemical equation representing aspirin hydrolysis. I) Arylesterase activity in fecal samples from MR and MD groups (n = 5). J) The ability to hydrolyze aspirin into salicylic acid of fecal samples from the MR and MD groups (n = 5). K–M) LC–MS detection of aspirin (K) and salicylic acid (L) in intestinal contents, and aspirin concentration in serum (M) of MR and MD mice 2 h after oral gavage of aspirin (2 mmol kg^−1^) (n = 5). *p* values were determined using the Mann‐Whitney *U* test, two‐tailed unpaired *t*‐test, one‐way ANOVA, or two‐way ANOVA. ns indicates no significant difference; ^**^ *p* value < 0.01; ^***^ *p* value < 0.001.

The effect of aspirin‐induced microbiota alterations was first examined by transplanting fecal microbiota from aspirin‐ or vehicle‐treated mice into recipients. Although 16S rRNA sequencing revealed that aspirin treatment significantly altered the microbial composition (Figure S2, Supporting Information), no significant differences were observed in histological intestinal injury, epithelial integrity, or permeability between recipient mice transplanted with aspirin‐altered microbiota and those with control microbiota (**Figure** **2**A–E). Therefore, aspirin‐mediated microbiota alteration does not appear to be associated with intestinal injury, suggesting that microbiota‐mediated aspirin biotransformation may be responsible for increased aspirin‐induced intestinal injury.

To validate this hypothesis, Caco‐2 cells were treated with bacteria‐free supernatants from aspirin incubated with MR or MD fecal microbiota (Figure 2F). Cells exposed to the aspirin–MR supernatant showed markedly reduced ZO‐1 expression compared with those treated with the aspirin–MD supernatant (Figure 2G). This confirms that microbiota‐mediated aspirin biotransformation contributes to aspirin‐induced intestinal injury. To elucidate how gut microbiota biotransform aspirin prior to its rapid absorption in the intestine, it was proposed that aspirin's most labile structure—the ester bond—is targeted by gut microbiota, resulting in enhanced hydrolysis into salicylic acid and acetic acid (Figure 2H). This hypothesis was supported by the observation that the MR mouse microbiota, compared with that of MD mice, markedly enhanced ester hydrolysis and promoted the conversion of aspirin to salicylic acid, as evidenced by arylesterase activity and the Fe(III)‐enhanced colorimetric assay (Figure 2I,J). To further directly verify the ability of gut microbiota to hydrolyze aspirin into salicylic acid, MR and MD mice were orally administered aspirin, and the levels of aspirin and salicylic acid in intestinal contents, as well as serum aspirin concentrations, were measured 2 h after gavage. The results showed that aspirin levels in the intestinal contents and serum of MR mice were significantly lower than those in MD mice, whereas salicylic acid levels in the intestinal contents were markedly higher in MR mice (Figure 2K–M). Collectively, these findings indicate that the gut microbiota promote the hydrolysis of aspirin into salicylic acid and acetic acid, which may underlie the observed differences in intestinal injury between the groups.

### 2.3 Salicylic Acid Hydrolyzed from Aspirin Promotes Intestinal Injury

**Figure 3:**
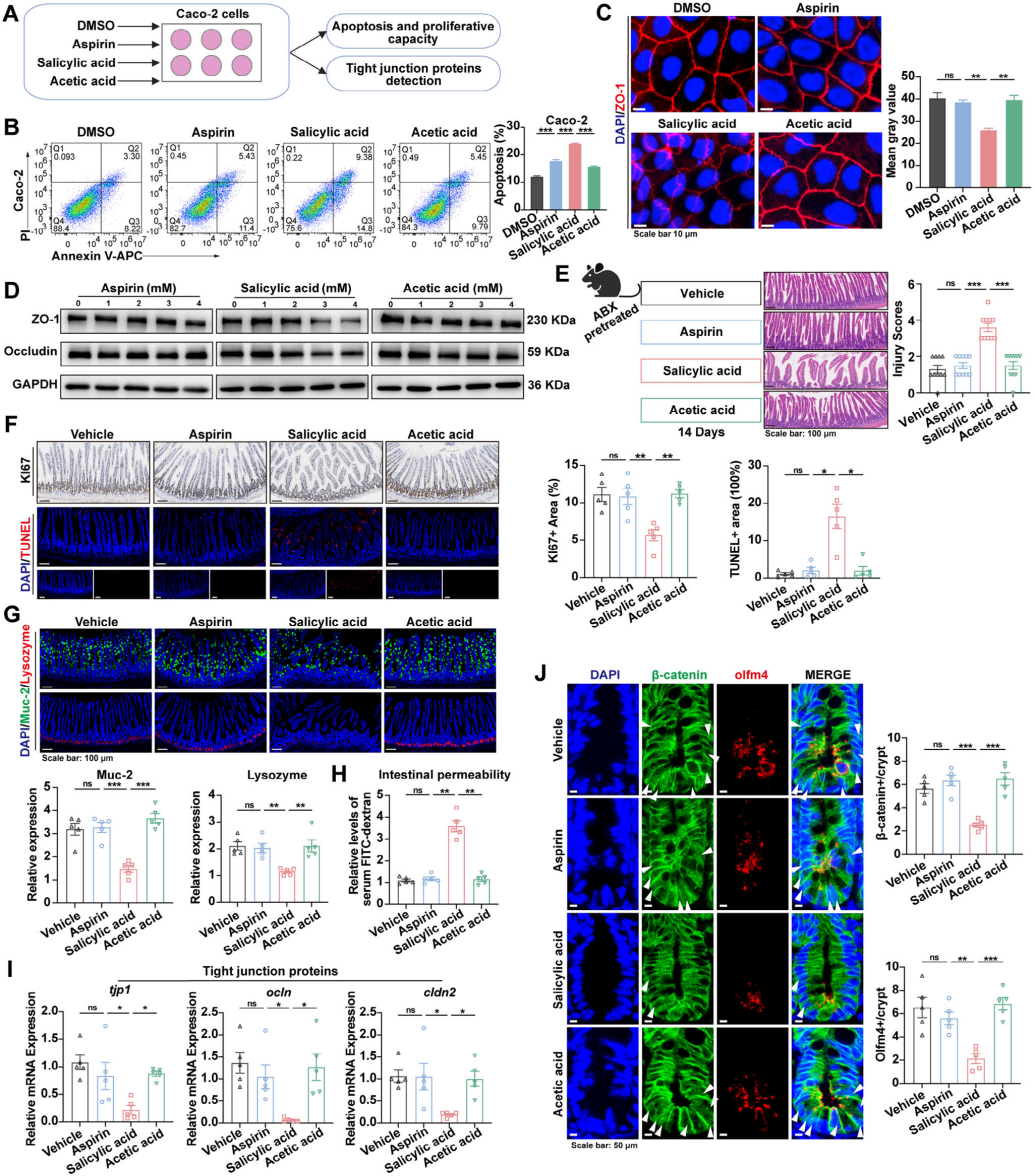
Salicylic acid hydrolyzed from aspirin promotes intestinal injury. A) Schematic diagram of Caco‐2 cells treated with equimolar concentrations of DMSO, aspirin, salicylic acid, or acetic acid. B) Flow cytometry analysis of Caco‐2 cell apoptosis after treatment with DMSO, aspirin, salicylic acid, or acetic acid (8 mM) (n = 3). C) Immunofluorescence staining of ZO‐1 expression in Caco‐2 cells treated with DMSO, aspirin, salicylic acid, or acetic acid (4 mM). D) Western blot analysis of Occludin and ZO‐1 protein expression in Caco‐2 cells. E) Schematic of the experimental design (left). H&E images and injury scores (right) (n = 10). F) Ki67 and TUNEL staining images (n = 5). G) Immunofluorescence of Muc2 (green) and lysozyme (red) (n = 5). H) Serum levels of FITC‐dextran (n = 5). I) Expression of *tjp1*, *ocln*, and *cldn2* in the small intestine (n = 5). J) Immunofluorescence images of β‐catenin (nuclear β‐catenin indicated by arrows) and Olfm4 (n = 5). *p* values were calculated using one‐way ANOVA or Kruskal–Wallis test. ns indicates no significant difference; ^*^ *p* < 0.05; ^**^ *p* < 0.01; ^***^ *p* < 0.001.

To assess the effects of aspirin and its hydrolysis products–salicylic acid and acetic acid–on intestinal injury, Caco‐2 cells were treated with equimolar concentrations of each compound (**Figure** **3**A). Salicylic acid was found to significantly increase apoptosis in Caco‐2 cells compared with aspirin or acetic acid (Figure 3B). Moreover, with increasing drug doses, salicylic acid exhibited a more potent inhibitory effect on cell proliferation than aspirin and acetic acid (Figure S3A, Supporting Information). Consistent with these results, higher concentrations of salicylic acid led to greater reductions in ZO‐1 and Occludin protein levels compared to aspirin and acetic acid (Figure 3C,D).

To substantiate the in vitro findings in vivo, mice were assessed for intestinal injury after oral administration of equimolar concentrations of aspirin, salicylic acid, or acetic acid for two weeks. To minimize the influence of gut microbiota on drug metabolism, mice were pretreated with antibiotics to deplete intestinal bacteria (Figure 3E). No significant liver or kidney damage was observed in mice across the different groups (Figure S3B–D, Supporting Information). Compared to mice treated with aspirin or acetic acid, those treated with salicylic acid exhibited increased histological intestinal injury (Figure 3E; Figure S3E, Supporting Information), decreased epithelial cell viability (Figure 3F), impaired barrier function (Figure 3G), enhanced permeability (Figure 3H), compromised epithelial integrity (Figure 3I), and reduced regenerative capability (Figure 3J). Together, these findings demonstrate that intestinal injury caused by salicylic acid, a hydrolysis product of aspirin, is more severe than that caused by aspirin itself.

Overall, the exacerbation of aspirin‐induced intestinal injury by gut microbiota is attributable to their role in promoting the hydrolysis of aspirin into salicylic acid, which subsequently intensifies intestinal damage.

### 2.4 *Lysinibacillus* Genus Facilitates Hydrolysis of Aspirin

**Figure 4:**
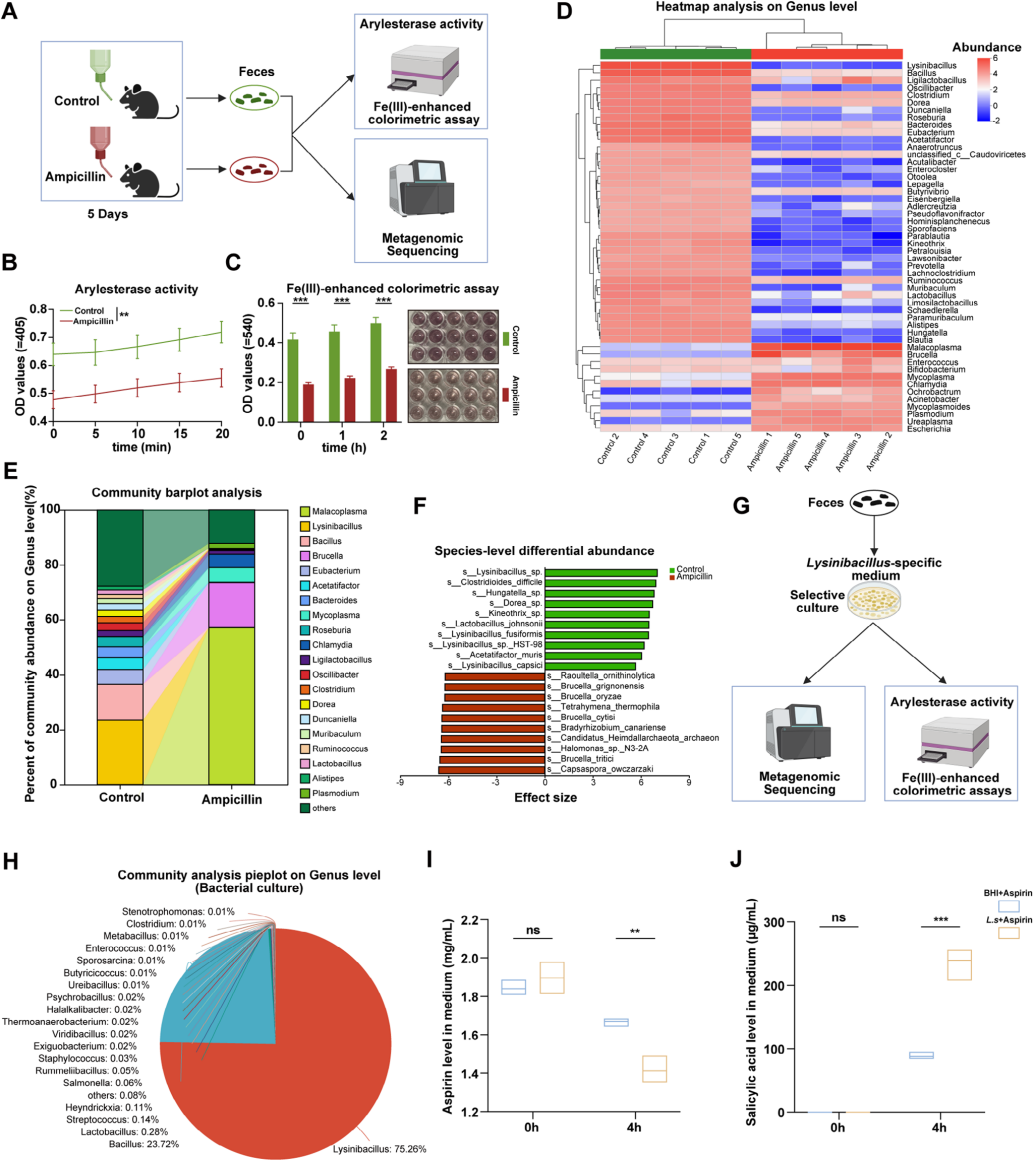
*Lysinibacillus* genus facilitates the hydrolysis of aspirin. A) Experimental workflow schematic. Created with BioRender.com. B) To evaluate the effect of ampicillin on fecal arylesterase activity in mice (n = 5). C) Effect of ampicillin on mouse fecal hydrolysis of aspirin to salicylic acid (n = 5). D) Heatmap analysis of the microbial community composition (n = 5). E) Comparative community bar plot analysis (n = 5). F) ALDEx2 analysis of microbial composition at the species level (n = 5). G) Selective culture of the genus *Lysinibacillus*. Created with BioRender.com. H) Community composition analysis of bacterial suspensions cultured in *Lysinibacillus*‐specific medium. I,J) Aspirin (2 mg mL^−1^) was incubated with *L. sphaericus*, and aspirin (I) and salicylic acid (J) levels in the culture supernatant were determined at 0 h and 4 h, using BHI medium as the control (n = 3). *p* values were calculated using a two‐way ANOVA test. ns indicates no significant difference; ^*^ *p* < 0.05; ^**^ *p* < 0.01; ^***^ *p* < 0.001.

Our findings showed that gut microbiota from MR mice exhibited higher arylesterase activity and a greater capacity to hydrolyze aspirin into salicylic acid compared to microbiota from MD mice (Figure 2I–M), indicating that in vivo antibiotic cocktail treatment inhibited bacteria with high arylesterase activity. In vitro experiments further confirmed that an antibiotic cocktail markedly suppressed arylesterase activity in fecal microbiota from antibiotic‐naive mice, with ampicillin alone producing a stronger effect than the other three antibiotics (Figure S4A, Supporting Information). On this basis, ampicillin was used in place of the cocktail for mouse treatment, which may offer advantages in narrowing the scope of key bacteria interrogation—specifically enabling the identification of those with high arylesterase activity that facilitate aspirin hydrolysis (**Figure** **4**A). Ampicillin significantly reduced the arylesterase activity and aspirin‐hydrolyzing capacity of fecal microbiota from antibiotic‐naive mice (Figure 4B,C). Metagenomic sequencing revealed that the fecal microbiota of ampicillin‐treated mice showed the most pronounced decline in the abundance of the *Lysinibacillus* genus (Figure 4D–F; Figure S4B, Supporting Information), suggesting this genus may play a critical role in aspirin hydrolysis.

To verify this hypothesis, enrichment of this genus was achieved by culturing fecal microbiota from antibiotic‐naive mice in a *Lysinibacillus*‐specific medium (Figure 4G). Metagenomic profiling confirmed the dominant presence of *Lysinibacillus* in the specific culture (Figure 4H; Figure S4C, Supporting Information). The *Lysinibacillus*‐enriched culture exhibited markedly higher arylesterase activity and a greater capacity for aspirin hydrolysis compared to the control medium (Figure S4D, Supporting Information). Among the species enriched within the *Lysinibacillus* genus, the potential functional roles of three representative strains were further validated: *Lysinibacillus fusiformis* (*L. fusiformis*), *Lysinibacillus sphaericus* (*L. sphaericus*), and *Lysinibacillus macrolides* (*L. macrolides*). The results showed that *L. sphaericus* exhibited the highest arylesterase activity and facilitated aspirin hydrolysis to the greatest extent (Figure S4E, Supporting Information). Furthermore, after incubation of *L. sphaericus* or BHI medium with aspirin for 4 h, LC‐MS analysis revealed that the aspirin concentration was markedly lower, while the salicylic acid concentration was significantly higher in the *L. sphaericus* culture than in the BHI medium (Figure 4I,J). These findings collectively indicate that *L. sphaericus* can hydrolyze aspirin into salicylic acid.

### 2.5 *Lysinibacillus Sphaericus* Amplifies Aspirin‐Induced Intestinal Injury In Vivo and In Vitro

**Figure 5:**
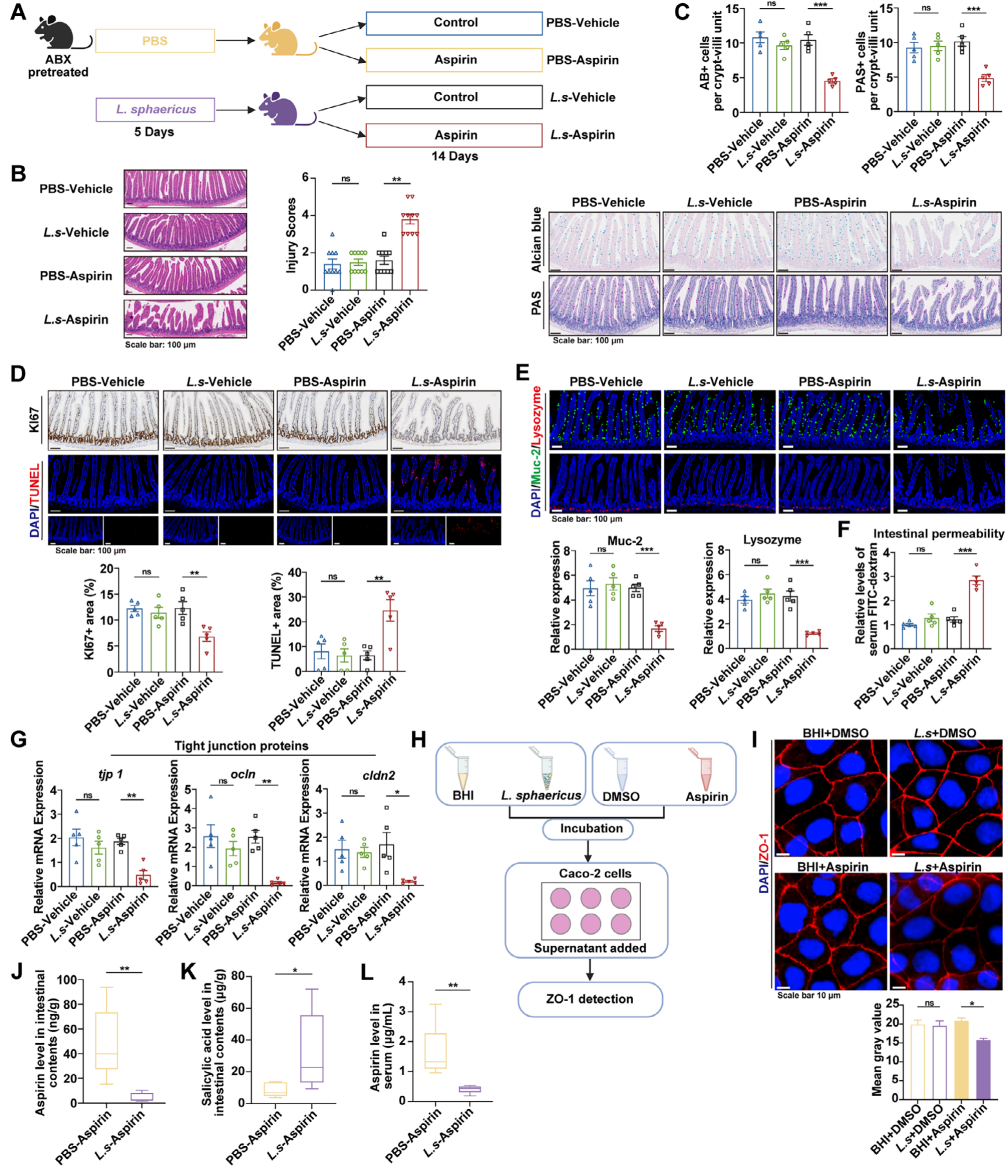
*Lysinibacillus sphaericus* amplifies aspirin‐induced intestinal injury in vivo and in vitro. A) Schematic of *L. sphaericus* colonization and aspirin‐induced intestinal injury model. Created with BioRender.com. B) H&E images and injury scores (n = 10). C) Alcian blue and PAS images (n = 5). D) Ki67 and TUNEL staining images (n = 5). E) Immunofluorescence of Muc2 (green) and lysozyme (red) (n = 5). F) Serum levels of FITC‐dextran (n = 5). G) Expression of *tjp1*, *ocln*, and *cldn2* in the small intestine (n = 5). H) Schematic diagram of cell experiment. Created with BioRender.com. I) Immunofluorescence of ZO‐1 expression in aspirin‐treated Caco‐2 cells with 5% *L. sphaericus* (n = 3). J–L) LC–MS analysis of aspirin (J) and salicylic acid (K) in intestinal contents, and (L) serum aspirin concentration 2 h after oral gavage of aspirin (2 mmol kg^−1^) in mice pretreated with *L. sphaericus* or PBS (n = 5). *p* values were determined by one‐way ANOVA and Kruskal–Wallis test. ns indicates no significant difference; ^*^ *p* < 0.05; ^**^ *p* < 0.01; ^***^ *p* < 0.001.

To evaluate the impact of *L. sphaericus* on aspirin‐induced intestinal injury, mice were orally administered *L. sphaericus* before evaluation of intestinal damage (**Figure** **5**A). qPCR analysis confirmed successful colonization of *L. sphaericus* in the intestinal tract (Figure S5A, Supporting Information). 16S rRNA sequencing of fecal microbiota confirmed that *L. sphaericus* colonization did not alter microbial diversity or overall composition compared to control mice (Figure S5B–D, Supporting Information). Notably, pathway analysis revealed a significant increase in drug metabolism in the *L. sphaericus*‐colonized mice (Figure S5E, Supporting Information). While colonization did not cause obvious intestinal damage relative to controls, it markedly worsened histological injury after aspirin treatment (Figure 5B,C), indicating that *L. sphaericus* plays a key role in exacerbating aspirin‐induced intestinal injury. Supporting this, *L. sphaericus‐*colonized mice exhibited reduced epithelial cell viability, impaired barrier function, increased permeability, and compromised epithelial integrity following aspirin administration (Figure 5D–G).

To assess the in vitro effects, Caco‐2 cells were exposed to bacteria‐free supernatants in which aspirin had been incubated with 5% *L. sphaericus* (Figure 5H). This treatment markedly reduced ZO‐1 expression in Caco‐2 cells, whereas *L. sphaericus* alone had no significant effect (Figure 5I). Furthermore, the levels of aspirin in the intestinal contents and serum of *L. sphaericus*‐colonized mice were significantly lower than those in PBS‐treated mice, while salicylic acid levels in the intestinal contents were markedly elevated (Figure 5J–L). Collectively, these results demonstrate that *L. sphaericus* not only promotes aspirin hydrolysis but also exacerbates aspirin‐induced intestinal injury.

### 2.6 Carboxylesterase EstB Secreted by *Lysinibacillus Sphaericus* Facilitates Aspirin Hydrolysis

**Figure 6:**
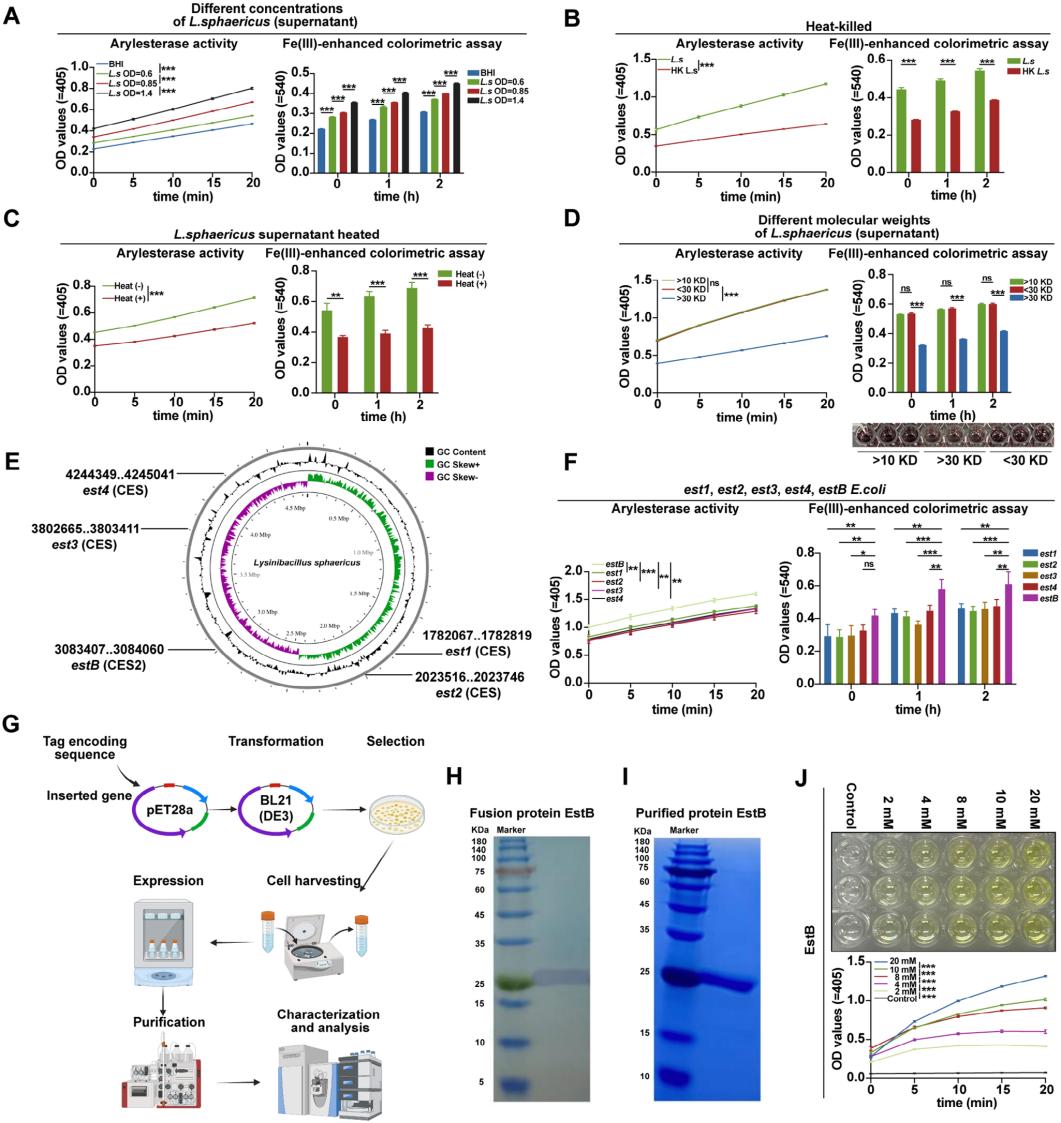
Carboxylesterase EstB secreted by *Lysinibacillus sphaericus* facilitates aspirin hydrolysis. A–D) Assessment of arylesterase activity and the ability to hydrolyze aspirin into salicylic acid of *L. sphaericus* and its supernatant following various treatments (n = 3). E) Circular genomic map of *L. sphaericus* generated using DNAPlotter. F) Arylesterase activity and the ability to hydrolyze aspirin into salicylic acid with *E. coli* overexpressing *est1, est2, est3, est4* or *estB* (n = 3). G) Flowchart illustrating the process of heterologous recombination, subsequent protein expression, and purification. Created with BioRender.com. H) Western blot analysis of the fusion EstB protein. I) SDS‐PAGE analysis of the purified EstB protein. J) Arylesterase activity of purified EstB (n = 3). *p* values were determined by a two‐way ANOVA test. ns indicates no significant difference; ^*^ *p* < 0.05; ^**^ *p* < 0.01; ^***^ *p* < 0.001.

Next, the mechanisms underlying *L. sphaericus*–mediated aspirin hydrolysis were investigated. Arylesterase activity and aspirin‐hydrolyzing capacity increased proportionally with bacterial concentration in both the supernatant and bacterial suspension (**Figure** **6**A; Figure S6A, Supporting Information), indicating a functional contribution of extracellular components. Moreover, these activities were significantly diminished in heat‐treated and repeatedly freeze‐thawed suspensions (Figure 6B; Figure S6B,C, Supporting Information), further demonstrating that the active extracellular components are secreted by live bacteria.

Extended analysis demonstrated that arylesterase activity and aspirin‐hydrolyzing capacity in the supernatant were significantly reduced following treatment at 95 °C, and the highest activity was observed at 37 °C compared to 4 and 65 °C (Figure 6C; Figure S6D, Supporting Information), suggesting that the active components are temperature‐sensitive. Notably, both activities were markedly inhibited by benzyl‐2‐nitrophenyl phosphate (BNPP) and zinc chloride (ZnCl_2_), which are known enzyme inhibitors (Figure S6E,F, Supporting Information); however, they were unaffected by DNase or RNase (Figure S6G,H, Supporting Information). Additionally, molecular sieve assays revealed that arylesterase activity was primarily enriched in the 10–30 kDa fraction, compared with <10 kDa or >30 kDa fractions (Figure 6D).

Given that experimental evidence indicates that the active components secreted by *L. sphaericus* are likely protein enzymes, it was hypothesized that hydrolases may play a key role in promoting aspirin hydrolysis. To explore this, genomic analysis was performed to identify genes potentially encoding hydrolases, as listed in Table S1 (Supporting Information). Intriguingly, only five genes encoding carboxylesterases—*est1*, *est2*, *est3*, *est4*, and *estB*—were identified in the *L. sphaericus* genome (Figure 6E), with no other hydrolase genes detected. ELISA assays confirmed significantly higher levels of carboxylesterases in *L. sphaericus* culture medium and in fecal microbiota from antibiotic‐naive mice, compared with controls or antibiotics‐treated mice, respectively (Figure S7A,B, Supporting Information). Notably, treatment with loperamide, a carboxylesterase inhibitor, markedly suppressed the arylesterase activity and aspirin‐hydrolyzing capacity of *L. sphaericus* (Figure S7C, Supporting Information). These findings collectively imply that carboxylesterases produced by *L. sphaericus* mediate its role in promoting aspirin hydrolysis.

To investigate the activity of the carboxylesterase‐encoding genes in *L. sphaericus*, each gene was overexpressed in *Escherichia coli* (*E. coli*). Among them, the strain overexpressing *estB* exhibited the highest arylesterase activity and the greatest capacity for aspirin hydrolysis compared with strains expressing the other four genes (Figure 6F), indicating that EstB from *L. sphaericus* is a major contributor to aspirin hydrolysis. To directly assess the role of EstB, a plasmid was constructed for *estB* transfection, and the recombinant EstB protein was subsequently overexpressed and purified (Figure 6G; Figure S7D, Supporting Information). The purified EstB had a molecular weight of ≈25 kDa (Figure 6H,I), consistent with the 10–30 kDa size range identified in molecular sieve assays (Figure 6D). EstB concentration positively correlated with both arylesterase activity and aspirin hydrolysis (Figure 6J; Figure S7E, Supporting Information), further confirming that EstB is a key driver of aspirin hydrolysis.

### 2.7 *Lysinibacillus Sphaericus*‐Derived EstB Deteriorates Aspirin‐Induced Intestinal Injury

To assess the impact of *L. sphaericus*‐derived EstB on aspirin‐induced intestinal injury, mice were orally administered *E. coli* carrying an empty vector (vector‐*E. coli*) or *estB*‐overexpressing *E. coli* (*estB‐E. coli*) for five days prior to aspirin treatment (Figure S8A, Supporting Information). To evaluate the colonization and functional activity of *estB‐E. coli* in the mouse gut, plasmid‐bearing bacteria were quantified in fecal samples. CFU enumeration on kanamycin‐containing BHI plates confirmed partial colonization of the gavaged strain. Consistently, qPCR analysis showed a marked increase in *estB* gene abundance in feces from the experimental group compared with the vector‐control group. Moreover, ELISA measurement of carboxylesterase levels in feces revealed significantly higher enzymatic activity in the *estB*‐overexpressing group, indicating that the engrafted *E. coli* were functionally active in vivo (Figure S8B, Supporting Information). Remarkably, *estB‐E. coli* administration markedly exacerbated aspirin‐induced intestinal injury, resulting in more severe histological damage, decreased epithelial cell viability, increased intestinal permeability, and compromised barrier integrity relative to vector‐*E. coli* (Figure S8C–H, Supporting Information). No significant injury was observed in aspirin‐naive mice, indicating that *estB‐E. coli* alone does not damage the intestine. These results indicate that *L. sphaericus*‐derived EstB does not directly harm the intestine but exacerbates aspirin‐induced injury.

### 2.8 Inhibition of *Lysinibacillus Sphaericus*‐Derived EstB Attenuates Aspirin‐Induced Intestinal Injury

**Figure 7:**
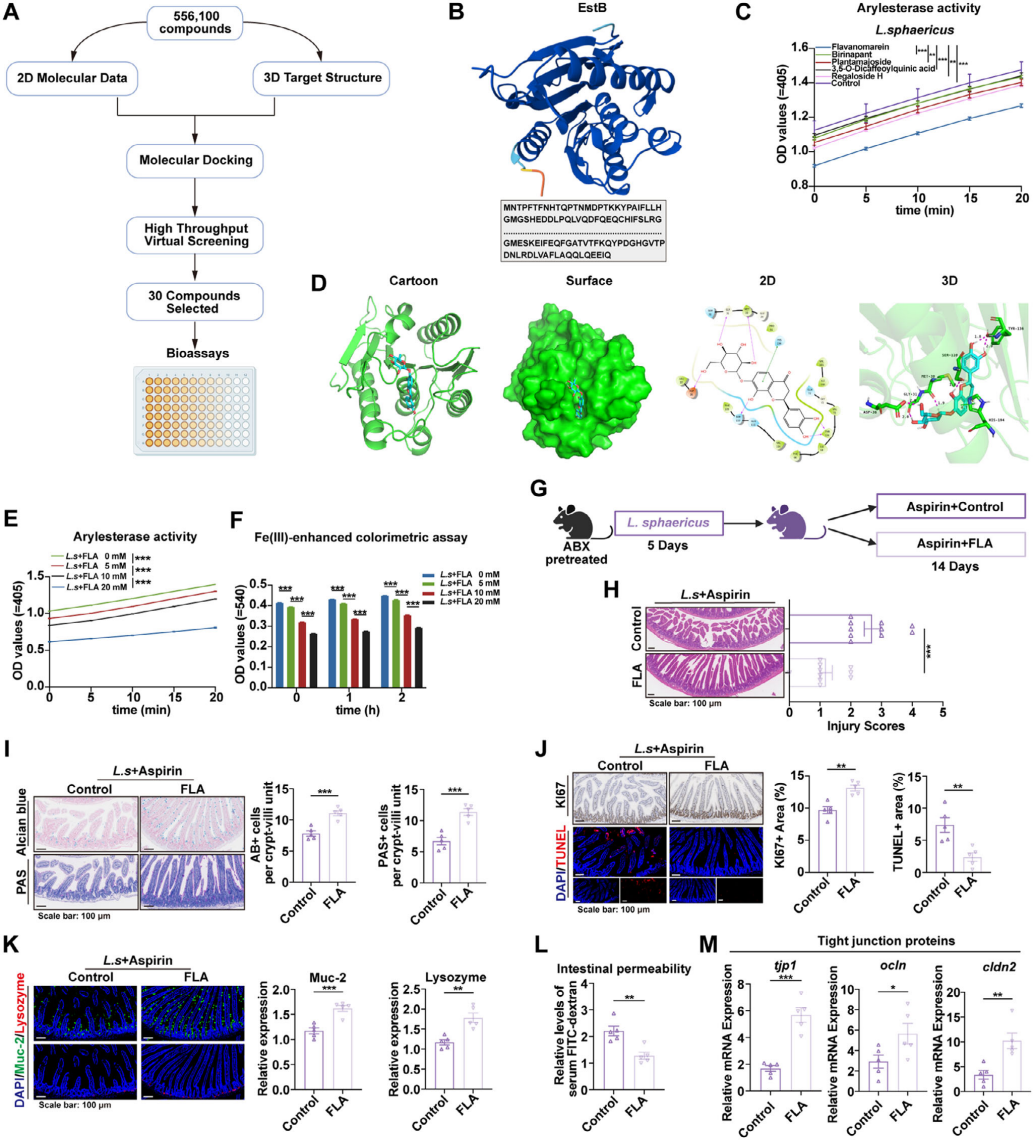
Inhibition of *L. sphaericus*‐derived EstB attenuates aspirin‐induced intestinal injury. A) Workflow of virtual screening based on molecular docking for 55610 compounds from the MedChemExpress (MCE) library. Created with BioRender.com. B) 3D structure of EstB. C) Arylesterase activity of the five most potent inhibitors (n = 3). D) The binding modes of FLA with EstB were visualized in cartoon, surface, 2D, and 3D representations. E) The effect of FLA on arylesterase activity of *L. sphaericus* (n = 3). F) The effect of FLA on the ability of *L. sphaericus* to hydrolyze aspirin into salicylic acid (n = 3). G) Schematic diagram of the animal experiment on the effect of FLA on aspirin‐related intestinal injury induced by *L. sphaericus*. Created with BioRender.com. H) H&E images and injury scores (n = 10). I) Alcian blue and PAS images (n = 5). J) Ki67 and TUNEL staining images (n = 5). K) Immunofluorescence of Muc2 (green) and lysozyme (red) (n = 5). L) Serum levels of FITC‐dextran (n = 5). M) Expression of *tjp1*, *ocln*, and *cldn2* in the small intestine (n = 5). *p* values were determined by Mann‐Whitney *U*, two‐way ANOVA, and two‐tailed unpaired *t*‐test. ^*^ *p* < 0.05; ^**^ *p* < 0.01; ^***^ *p* < 0.001. FLA: flavanomarein.

Since *L. sphaericus*‐derived EstB mediates aspirin‐induced intestinal injury, specific inhibitors were sought to modulate this process. Based on structural analysis of EstB (**Figure** **7**A,B), 556 100 compounds from the MedChemExpress (MCE) library were subjected to virtual screening. The top 30 candidates, ranked by binding scores, were selected for validation by measuring arylesterase activity in media co‐incubated with *L. sphaericus* (Figure S9A and Data File S1, Supporting Information). Among these, flavanomarein was identified as the most effective inhibitor, markedly reducing arylesterase activity to the greatest extent (Figure 7C). Its binding mode with *L. sphaericus*‐derived EstB was visualized using cartoon, surface, 2D, and 3D representations (Figure 7D). Flavanomarein exhibited dose‐dependent inhibition of both the arylesterase activity of *L. sphaericus* and its capacity to hydrolyze aspirin (Figure 7E,F). In vivo, flavanomarein significantly attenuated histological intestinal injury, improved epithelial cell viability, enhanced barrier function, reduced epithelial permeability, and preserved epithelial integrity in aspirin‐treated mice colonized with *L. sphaericus* (Figure 7G–M). To determine whether flavanomarein exerts its protective effects specifically by inhibiting aspirin hydrolysis, rather than by broadly preventing intestinal injury, it was administered in a salicylic acid‐treated mouse model. Flavanomarein failed to alleviate intestinal injury induced by salicylic acid (Figure S9B–D, Supporting Information). Moreover, in the *L. sphaericus* + aspirin group, flavanomarein treatment led to reduced carboxylesterase levels (Figure S9E, Supporting Information), higher aspirin concentrations in both intestinal contents and serum, and lower salicylic acid levels in intestinal contents compared with the same group without flavanomarein (Figure S9F, Supporting Information). Collectively, these findings demonstrate that flavanomarein inhibits *L. sphaericus*‐mediated aspirin hydrolysis and alleviates aspirin‐induced intestinal injury, further confirming the causal role of EstB in facilitating aspirin hydrolysis and exacerbating aspirin‐associated enterotoxicity.

## 3 Discussion

It is widely accepted that aspirin‐induced gastrointestinal injury is primarily attributed to the inhibition of cyclooxygenase‐1 (COX‐1), leading to reduced prostaglandin synthesis. This mechanism also explains the markedly lower gastrointestinal irritation observed with COX‐2 selective inhibitors.^[^ ^20^ ^]^ However, animal studies have shown that COX‐1‐deficient mice do not spontaneously develop gastrointestinal ulcers but still exhibit ulceration or bleeding when exposed to aspirin or other NSAIDs,^[^ ^21^ ^]^ suggesting that additional COX‐1‐independent mechanisms may contribute to aspirin‐induced enterotoxicity. Here, we demonstrate that gut microbiota exacerbate aspirin‐induced intestinal injury by enhancing aspirin conversion to salicylic acid, a biotransformed isoform with greater enterotoxicity than the parent compound. Specifically, the genus *Lysinibacillus*, particularly *L. sphaericus*, shows remarkable activity in facilitating aspirin conversion to salicylic acid. The carboxylesterase EstB encoded by *L. sphaericus* is responsible for aspirin‐to‐salicylic acid hydrolysis. This key enzyme can be effectively inhibited by flavanomarein, thereby reducing the hydrolysis of aspirin into salicylic acid and alleviating aspirin‐induced enterotoxicity.

NSAIDs‐induced mucosal injury has been linked to the uncoupling of mitochondrial oxidative phosphorylation,^[^ ^22^ ^]^ a process negatively associated with the drugs’ logarithmically transformed acid dissociation constant (pKa).^[^ ^23^ ^]^ Salicylic acid, with a lower pKa (2.98) than aspirin (3.5), may more potently induce injury by promoting mitochondrial uncoupling.^[^ ^24^ ^]^ In general, lower pKa values correspond to stronger acidity, whereas non‐acidic pro‐NSAIDs, such as nabumetone and nitro‐butyril flurbiprofen, are considered safer for the mucosa.^[^ ^25^, ^26^ ^]^ Historically, salicylic acid was derived from salicin in medicinal willow bark; its gastrointestinal irritation prompted the development of acetylated aspirin, which improved tolerability.^[^ ^27^ ^]^ Together, these findings and historical context support the conclusion that microbiota‐mediated hydrolysis of aspirin to salicylic acid is a key driver of aspirin‐induced intestinal injury.

Previous evidence shows that antibiotic treatment with ampicillin markedly elevates circulating aspirin levels following oral administration,^[^ ^18^, ^19^ ^]^ implying that ampicillin‐depleted bacteria have a substantial capacity to degrade aspirin before its absorption into the bloodstream. In agreement with this, we observed that among the four antibiotics in the cocktail, only ampicillin most effectively suppressed microbiota‐mediated hydrolysis of aspirin in vitro. Metagenomic sequencing revealed a notable reduction of the genus *Lysinibacillus* following ampicillin treatment. Through screening of representative species within this genus, we identified *L. sphaericus* as the species with the greatest capacity to hydrolyze aspirin into salicylic acid. Mice colonized with *L. sphaericus* exhibited enhanced aspirin‐induced intestinal injury, while controls without aspirin treatment showed no such effect (Figure 5). These results suggest that *L. sphaericus* does not cause toxicity directly but exacerbates injury by facilitating aspirin hydrolysis.

*L. sphaericus* is a Gram‐positive, spore‐forming aerobe from the *Bacillaceae* family.^[^ ^28^ ^]^ Its strict aerobic metabolism makes it well‐adapted for growth and activity in the relatively oxygen‐rich environment of the proximal small intestine,^[^ ^29^, ^30^ ^]^ where aspirin absorption predominantly occurs.^[^ ^31^ ^]^ Evidence from high‐altitude rat models shows that reduced ambient oxygen levels are associated with increased aspirin absorption; furthermore, fecal microbiota suspensions from these rats exhibit diminished aspirin metabolism.^[^ ^32^ ^]^ This supports that aerobic bacteria—such as *L. sphaericus*—within the gut microbiota may contribute to aspirin metabolism. Generally, aspirin can undergo reversible hydrolysis to salicylic acid and acetic acid, which may re‐synthesize back into aspirin. Interestingly, *L. sphaericus* does not metabolize carbohydrates but instead utilizes acetate or glycerol as carbon sources.^[^ ^33^ ^]^ This implies that it might consume acetic acid and shift aspirin hydrolysis from a reversible, bidirectional process into an irreversible, unidirectional one, thereby further promoting its conversion into salicylic acid.

Functional studies identified *L. sphaericus*‐derived carboxylesterase EstB as the key enzyme mediating aspirin hydrolysis and intestinal injury (Figure 6; Figures S6–S8, Supporting Information). Carboxylesterases, key members of the esterase family, catalyze the hydrolysis of various endogenous and exogenous ester‐containing compounds, thereby playing vital roles in drug activation and hydrolysis.^[^ ^34^, ^35^ ^]^ To date, six carboxylesterase‐encoding genes have been identified in humans and twenty in mice; among these, carboxylesterase‐1 (CES1) and carboxylesterase‐2 (CES2) are considered the most functionally significant due to their broad biological impact.^[^ ^36^ ^]^ CES1 primarily targets small alcohol‐large acyl esters, while CES2 prefers large alcohol‐small acyl substrates.^[^ ^37^ ^]^ Interestingly, EstB from *L. sphaericus* is a CES2‐like enzyme, a feature that mechanistically explains its enhanced hydrolytic activity toward aspirin, whose large aromatic alcohol group and small acetyl group favor CES2‐like substrate recognition.

Crucially, we demonstrate that flavanomarein, a potent inhibitor of *L. sphaericus‐*derived EstB, effectively mitigates aspirin‐induced intestinal injury, thereby confirming the causal role of EstB in exacerbating aspirin enterotoxicity (Figure 7). Flavanomarein belongs to the flavonoid family,^[^ ^38^ ^]^ a group of natural dietary phenolic compounds found in edible plants, widely used in nutraceuticals, pharmaceuticals, and medicine.^[^ ^39^ ^]^ Notably, recent studies have identified several flavonoids capable of inhibiting both CES1 and CES2,^[^ ^35^ ^]^ which aligns well with our findings.

**Figure 8:**
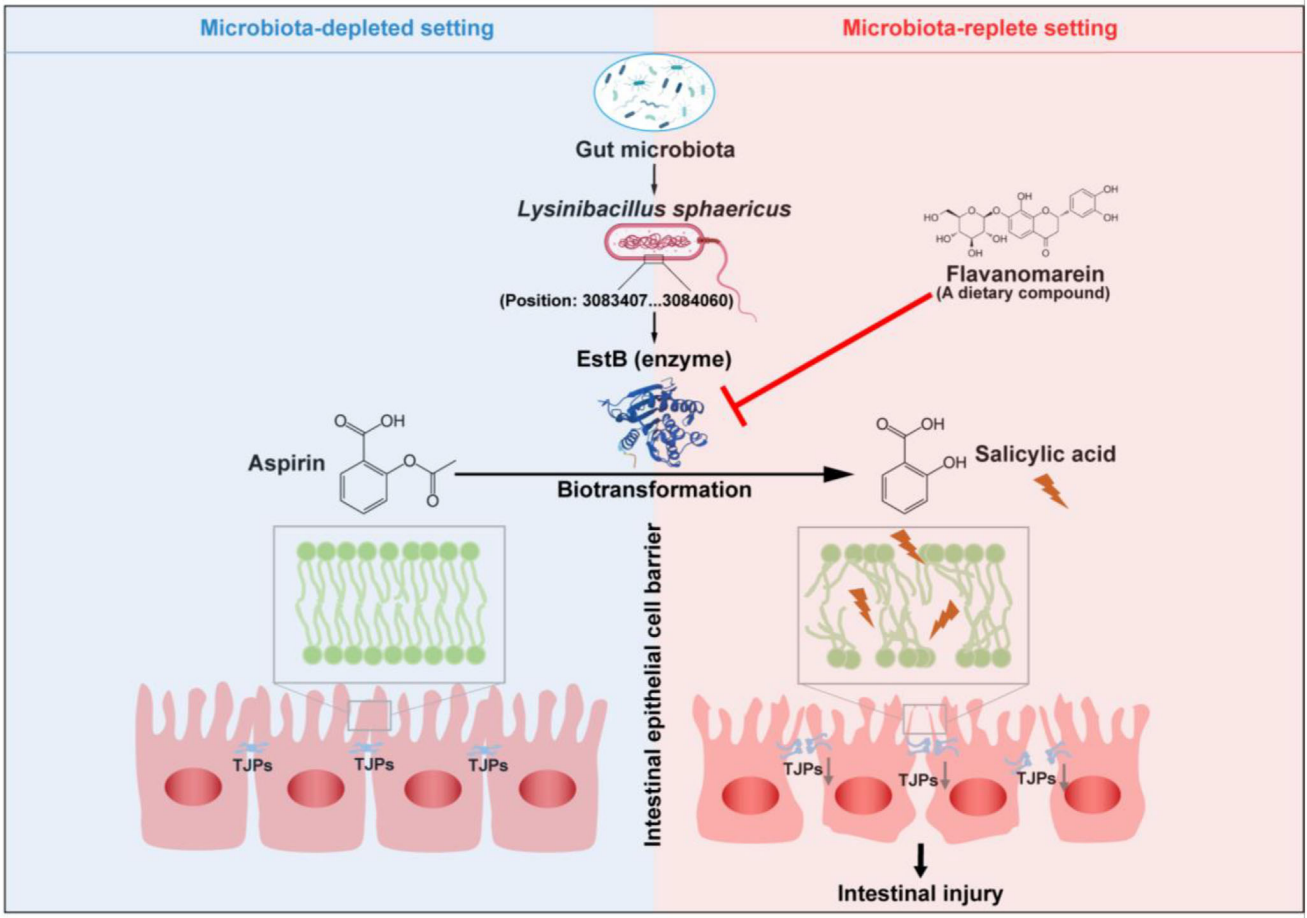
Graphical summary of the study. Schematic overview illustrating the detrimental role of gut microbiota in aspirin‐induced intestinal injury. *L. sphaericus* and its secreted carboxylesterase EstB were identified as key drivers that catalyze aspirin hydrolysis into salicylic acid, thereby exacerbating intestinal injury. Inhibition of EstB by the dietary compound flavanomarein effectively blocked aspirin biotransformation and alleviated intestinal toxicity, highlighting a potential microbiota‐targeted strategy for prevention.

This proof‐of‐concept study has several limitations. The role of gut microbiota in aspirin‐induced intestinal injury remains largely unexplored, and ethical and practical constraints make it infeasible to investigate this in humans. Here, we conducted a preclinical study to elucidate the contribution of gut microbiota, identifying *L. sphaericus* and its carboxylesterase EstB as key mediators. While further human studies are needed to confirm these findings, and other microbes or enzymes may also contribute to aspirin biotransformation, this work provides a foundation for understanding microbiota‐driven aspirin toxicity and for developing targeted interventions to prevent enterotoxicity. In conclusion, we provide the first demonstration that gut microbiota exacerbate aspirin‐induced intestinal injury by promoting its hydrolysis into salicylic acid in mice. Notably, *L. sphaericus* within the gut microbiota plays a pivotal role in amplifying this injury through the secretion of carboxylesterase EstB, an enzyme whose activity can be effectively inhibited by flavanomarein (**Figure** **8**).

## Conflict of Interest

The authors declare no conflict of interest.

## Author Contributions

Z.Z., Q.L., X.B., and E.Z. contributed equally to this work. Z.Y.Z. designed and conducted the key experiments, analyzed the data, and drafted the manuscript. Q.L., X.W.B., and E.T.Z. assisted in data acquisition, sample preparation, and experimental validation. W.G.D., Y.Q., T.H.Z., Z.X.H., Z.Y.H., and F.G.M. contributed to sample collection and technical support. S.R.C., T.Z., and J.H.C. provided input on data analysis and manuscript revision. R.S.Z. supervised the overall study, provided intellectual input and critical revision, and is responsible for the integrity of the work as the guarantor. All authors reviewed and approved the final version of the manuscript.

## Ethics Statement

All experimental protocols were approved by the IEC for Clinical Research and Animal Trials of the First Affiliated Hospital of Sun Yat‐sen University (Approval No. [2024]142).

## Supporting information



Supporting Information

## Data Availability

The data that support the findings of this study are available from the corresponding author upon reasonable request.
