# Supplementary material for: Gut Bacterium Lysinibacillus Sphaericus Exacerbates Aspirin‐induced Intestinal Injury by Production of Carboxylesterase EstB
Source: Adv Sci (Weinh). 2025 Dec 12;13(12):e17747. doi: 10.1002/advs.202517747 (PMC12948251; doi:10.1002/advs.202517747)
Supplement: Supplementary file 1 — Supporting Information [file ADVS-13-e17747-s001.docx]

Gut bacterium *Lysinibacillus sphaericus* exacerbates aspirin-induced intestinal injury by production of Carboxylesterase EstB

Zeyu Zhao^1,2†^, Qing Li^3†^, Xiaowu Bai^4†^, Ertao Zhai^1†^, Weigang Dai^1^, Yan Qian^1^, Tianhao Zhang^1^, Zhixin Huang^1^, Ziyu Huang^2,5^, Fangang Meng^6^, Jianhui Chen^1*^, Tao Zuo^2,5*^, Shirong Cai^1*^ & Risheng Zhao^1*^

**SUPPLEMENTARY MATERIALS AND METHODS**

**METHODS**

**Mice**

Six- to eight-week-old C57BL/6 mice were obtained from the Animal Center of the First Affiliated Hospital of Sun Yat-sen University. The mice were housed under standard SPF conditions, maintained at a controlled temperature (23 ± 2℃) with a strict 12-hour light/dark cycle, and were provided ad libitum access to food and water. All mice were randomly assigned to experimental groups and handled in accordance with the approved Institutional Animal Care and Use Committee (IACUC) protocols.

Antibiotic-mediated gut microbiota depletion: Mice in the antibiotics-treatment group received a combination of ampicillin (2 g/L), metronidazole (2 g/L), neomycin sulfate (2 g/L), and vancomycin (1 g/L) in their drinking water ad libitum for 5 days to deplete gut microbiota, followed by a 1-day recovery on sterile water. Mice in the ampicillin-only group were provided with drinking water containing ampicillin (2 g/L) ad libitum for 5 days.

Fecal microbiota transplantation (FMT): Recipient mice were pretreated with antibiotics to deplete intestinal microbiota, followed by oral gavage of 200 µL fecal suspension daily for 5 days before drug administration and twice weekly during the treatment period. For donor preparation, 200 mg of fresh feces were collected, suspended in 1 mL of phosphate-buffered saline (PBS), vortexed thoroughly, and centrifuged at 2,000 × g for 10 minutes at 4℃. The supernatant was mixed with an equal volume of 50% glycerol, aliquoted, and stored at -80℃ until use. To eliminate potential antibiotic residues, mice in the antibiotic-treated donor group continued to receive the antibiotic cocktail for five consecutive days and were then given sterilized drinking water for an additional three days before fecal collection, allowing sufficient time for antibiotic metabolism and minimizing residual effects on the FMT recipients.

Drug preparation: To minimize DMSO toxicity in mice and maintain drug stability, drugs were dissolved according to the methods recommended by MedChemExpress. All drugs were prepared as stock solutions, aliquoted, and stored at -80℃. Working solutions were freshly prepared using a solvent system of 10% DMSO, 40% PEG300, 5% Tween-80, and 45% saline.

Drug administration: Based on previous reports indicating a median lethal dose (LD_50_) of approximately 1,100 mg/kg for aspirin in mice^1^, and our preliminary experiments showing increased mortality at doses exceeding 5 mmol/kg (~900 mg/kg), a dose of 2 mmol/kg (~360 mg/kg) was selected for this study. Notably, this dose is lower than the mouse-equivalent dose of a typical human aspirin regimen (100 mg/60 kg/day), which corresponds to approximately 11.1 mg/20 g (~555 mg/kg)^2^. Similarly, salicylic acid and acetic acid were administered at 2 mmol/kg. All drugs were administered once daily via oral gavage for 14 consecutive days. Loperamide was administered at 10 mg/kg every other day for two weeks, and flavanomarein at 10 mg/kg daily for two weeks.

*L. sphaericus* colonization: Prior to gavage, *L. sphaericus* cultures were diluted in PBS to a final concentration of 1×10^9^ CFU/0.2 mL. Mice were pretreated with antibiotics and then administered the bacterial suspension by oral gavage once daily for 5 days before drug administration, followed by twice-weekly gavage during the treatment period to maintain stable colonization.

Gavage with *E. coli* in mice: To establish differential intestinal EstB enzyme levels, antibiotic-pretreated mice were orally administered either vector-*E. coli* or estB-*E. coli* (1×10^9^ CFU/0.2 mL) once daily for five consecutive days, followed by twice-weekly gavage during the drug treatment period. The *E. coli* strains were induced with 0.25 mM isopropyl β-D-1-thiogalactopyranoside (IPTG) prior to gavage to induce EstB expression. Carboxylesterase activity in fecal samples was measured to verify differential EstB activity between groups.

Sample collection: Intestinal contents were aseptically collected, flash-frozen in liquid nitrogen, and stored at -80℃. A 5 cm segment of the jejunum was fixed in formalin and prepared as a “Swiss roll” for histological analysis. For molecular studies, intestinal tissues were rinsed with ice-cold PBS to remove luminal contents, flash-frozen in liquid nitrogen, and stored at -80℃. Liver and kidney tissues were also collected and fixed in formalin. Blood samples were obtained via orbital sinus puncture; after clotting at room temperature for 1 hour, samples were centrifuged at 3,000 × g for 10 minutes to collect serum.

**Histological Analysis**

Formalin-fixed paraffin-embedded sections of small intestine ("Swiss roll" preparation), liver, and kidney tissues were stained with H&E. The histological assessment of intestinal damage was conducted using Chiu’s scoring system^3,4^. Intestinal injury was graded on a scale ranging from 0 (normal) to 5 (severe injury) as follows: 0-Normal intestinal villi without damage. 1-Formation of subepithelial spaces at the villus apex accompanied by capillary congestion. 2-Expansion of the submucosal space with elevation of the lamina propria due to edema. 3-Extensive epithelial detachment on both sides of the villi, degeneration and necrosis of mucosal epithelial cells, and partial loss of villus tips. 4-Severe degeneration, necrosis, and detachment of mucosal epithelium; partial villus shedding exposing the lamina propria; capillary dilation and congestion. 5-Complete villus loss, disintegration of the lamina propria, hemorrhage, and ulceration. For each mouse, at least five randomly selected areas of the intestine were scored. Histological scoring was performed independently and blindly by two pathologists. Paraffin-embedded intestinal sections were further subjected to PAS staining and Alcian blue staining to observe goblet cell distribution within the epithelial layer. Images were acquired using an automated digital pathology slide scanner (KF-PRO-020, Kfbio).

**Immunohistochemistry (IHC) & Immunofluorescence**

Swiss roll-embedded small intestine tissue sections were deparaffinized in xylene and rehydrated through a graded ethanol series. Antigen retrieval was performed using a citrate buffer solution. The sections were blocked with 10% goat serum at room temperature for 30 minutes and incubated overnight at 4℃ in a humidified chamber with primary antibodies, including anti-Ki-67 (12202, Cell Signaling Technology), anti-Olfm4 (39141, Cell Signaling Technology), anti-β-Catenin (8480, Cell Signaling Technology), anti-Muc2 (ab308191, Abcam), and anti-Lysozyme (ab108508, Abcam), as per the manufacturers' recommended dilutions. Immunohistochemistry was carried out using horseradish peroxidase-conjugated goat anti-rabbit IgG (31460, Invitrogen). For immunofluorescence, Alexa Fluor 488-conjugated goat anti-rabbit IgG (A-11008, Invitrogen) and Alexa Fluor 594-conjugated goat anti-rabbit IgG (A-11012, Invitrogen) were applied to detect fluorescence signals. Slides were mounted using DAPI-containing mounting medium (Abcam), and images were captured using a fluorescence slide scanner (DM6B, Leica).

For cell immunofluorescence, 5×10^4^ Caco-2 cells were seeded in each well of a 24-well plate containing coverslips. After 48 hours, cells were treated with bacteria or drugs for 24 hours, then fixed with paraformaldehyde. Permeabilization and blocking were performed with 10% goat serum containing 0.25% Triton X-100 for 30 minutes. Cells were incubated overnight at 4℃ with anti-ZO-1 (21773-1-AP, Proteintech), followed by incubation at room temperature with goat anti-rabbit Alexa Fluor 594-conjugated secondary antibody (A-11012, Invitrogen) for 1 hour in the dark. Coverslips were mounted on slides with DAPI-containing anti-fade mounting medium and observed under an inverted fluorescence microscope.

**TdT-Mediated dUTP Nick-End Labeling (TUNEL) Assay**

Cell death in tissues was assessed using an in situ cell death detection kit (Roche). Small intestine tissue sections were deparaffinized, rehydrated, and incubated in 0.1% citrate buffer containing 0.1% Triton X-100 for 15 minutes, followed by PBS washes. Samples were then incubated in TUNEL reaction mixture in a humidified chamber at 37℃ for 60 minutes in the dark. After washing with PBS, slides were mounted with DAPI-containing mounting medium (Solarbio). Images were acquired using a fluorescence slide scanner (DM6B, Leica).

**Intestinal Permeability Detection with Fluorescein-Isothiocyanate-Dextran**

Intestinal permeability was assessed using FITC-dextran. Mice were fasted for 4 hours and then administered FITC-dextran solution (0.4 mg/g body weight, Sigma) in sterile water. After 4 hours, blood was collected via orbital puncture and stored in the dark. Serum was obtained by centrifugation at 3,000 × g for 10 minutes at room temperature. A 20 µL aliquot of serum was diluted with PBS to 200 µL and placed into a 96-well plate alongside a FITC-dextran standard curve. Fluorescence was measured using a spectrophotometer at an excitation wavelength of 488 nm and an emission wavelength of 525 nm.

**Metabolite Extraction and Analysis**

Intestinal contents, serum, and culture supernatants were collected and extracted with acetonitrile to measure aspirin and salicylic acid levels. After centrifugation at 20,000 × g for 10 min, the supernatant was collected and subjected to UPLC-TQ-MS analysis (Waters ACQUITY UPLC I-Class System-SCIEX 6600 plus) equipped with an ACQUITY BEH C18 column (1.7 μm, 2.1 mm ID×100 mm, Waters) eluting at a flow rate of 0.4 mL/min. The mobile phase A was water/formic acid (100/0.1, v/v), and mobile phase B was acetonitrile/formic acid (100/0.1, v/v). The UPLC separations were 10 min/sample using the following scheme: (1) 0-1 min, 2% B; (2) 5 min, 98% B; (3) 5-7 min, 98% B; (4) 7.1 min, 2% B; (5) 7.1-10 min, 2% B. All the changes were linear. 2 μL samples were injected for analysis by mass spectrometer. Targeted metabolomics analysis of LC-MS data was performed using SCIEX OS 3.4.5 software.

**Arylesterase Activity Assay**

Arylesterase activity was assessed using 4-Nitrophenyl acetate as a substrate to evaluate hydrolysis capacity, indirectly reflecting the ability to hydrolyze aspirin^5^. Briefly, a 35 mmol/L stock solution was prepared by dissolving 63 mg of 4-Nitrophenyl acetate in 10 mL of methanol was prepared, and stored at -20℃. A 0.35 mmol/L working solution (freshly prepared) was prepared as the stock solution 1:100 with ddH_2_O. Add 190 μL of the working solution to each well of a 96-well plate, followed by 10 μL of fecal suspension or bacterial culture. Incubate at 37℃ for 30 minutes, and measure absorbance at 405 nm every 5 minutes using a multimode microplate reader for a total of 20 minutes.

**Salicylic Acid Assay**

The Fe(III)-enhanced colorimetric assay was used to measure salicylic acid, the primary metabolite of aspirin in the medium^6^. The color reagent solution was prepared by dissolving 8 g ferric nitrate, 8 g mercuric chloride, and 24 mL 1 N HCl in ddH_2_O to a final volume of 200 mL. To avoid exposure to the toxic chemicals, all procedures were carried out in a well-ventilated fume hood with appropriate protective equipment, and the resulting chemical waste was properly collected and disposed of according to institutional safety regulations. Equal volumes of fecal suspension or bacterial culture and aspirin (10 mM) were incubated at 37℃ for 2 hours. The mixture was then combined with solution at a 1:5 ratio, and 200 μL of the mixture was added to each well of a 96-well plate in triplicate. The plate was incubated at 37℃, and absorbance at 540 nm was measured at 0, 1, and 2 hours. Higher OD values indicate a greater concentration of salicylic acid.

**RNA Extraction and RT-PCR**

Total RNA was extracted from frozen small intestine tissues using an RNA rapid extraction kit (ES Science, Guangzhou, Guangdong, China). cDNA was synthesized from 2 µg of total RNA using the Evo M-MLV RT kit (Precision Biotechnology, Changsha, Hunan, China). Quantitative real-time PCR (RT-qPCR) was performed on a LightCycler 480 Real-Time PCR System (Roche, Basel, Switzerland) using the SYBR Green Premix Pro Taq HS qPCR kit (Accurate Biotechnology) following the manufacturer’s two-step protocol. GAPDH was used as the internal control. All reactions were run for 40 cycles, and relative mRNA levels were analyzed using Ct values. To quantify *L. sphaericus* in mouse feces, genomic DNA was prepared as described in the “DNA Extraction and Preparation” section. Absolute quantification by qPCR (40 cycles) was performed, and Ct values were converted to copies per gram of feces based on a standard curve generated from serial dilutions of *L. sphaericus* genomic DNA. All primers used are listed in Supplementary Table 2.

**Cell Culture**

Caco-2 cells were obtained from ATCC and cultured in DMEM supplemented with 10% FBS and 1% penicillin-streptomycin. The medium was refreshed every three days. When cell confluence reached approximately 70%, cells were passaged or seeded for experiments. Drug treatments were applied at ~70% confluence, and cells were harvested 24 hours post-treatment for apoptosis assays or protein extraction.

**Apoptosis Assay**

Caco-2 cells and supernatants were collected post-drug induction and washed three times with PBS. Apoptosis was assessed using the Annexin V-FITC/PI double staining apoptosis detection kit (KeyGEN, Jiangsu, China) following the manufacturer's instructions. Stained cells were analyzed by flow cytometry (Beckman, California, USA).

**Cell Viability Assay**

Each well of a 96-well plate was seeded with 200 µl of DMEM complete growth medium containing 2×10^4^ Caco-2 cells. After 48 hours, cells were treated with varying concentrations of aspirin, salicylic acid, or acetic acid. Following a 24-hour treatment, the medium was replaced with 100 µL of serum-free DMEM containing CCK-8 reagent (diluted 1:9). Cells were incubated at 37℃ for 2 hours in the dark, and absorbance was measured at 450 nm using a microplate reader.

**DNA Extraction and Preparation**

Mouse fecal DNA was extracted using the QIAamp PowerFecal Pro DNA Kit (QIAGEN, Germany). DNA degradation and contamination were assessed on 1% agarose gel, while purity and concentration were measured with a Nanophotometer (Implen, Munich, Germany). The extracted DNA was stored at -80℃ for further analysis.

**16S rRNA Gene Amplicon Sequencing**

Mouse fecal DNA was amplified for the V3-V4 region using universal primers 338F (5'-ACTCCTACGGGAGGCAGCAG-3') and 806R (5'-GGACTACHVGGGTWTCTAAT-3'). Amplicons were extracted from 2% agarose gel and purified using the AxyPrep DNA Gel Extraction Kit (Axygen Biosciences, Union City, CA, USA). Purified amplicons were pooled at equimolar concentrations and subjected to high-throughput sequencing on the Illumina platform.

**Metagenomic Sequencing**

Extracted DNA was fragmented to an average size of ~350 bp using the Covaris M220 system (GENEWIZ, China), and PE libraries were prepared using the NEXTFLEX Rapid DNA-Seq Kit (Bioo Scientific, Austin, TX, USA). Metagenomic sequencing was performed on the Illumina NovaSeq™ X Plus platform (Illumina, San Diego, CA, USA). Raw sequencing data were deposited in the NCBI SRA database. Data analysis was conducted via the free online Majorbio Cloud platform. Adapter trimming and quality control of paired-end reads were performed using fastp (version 0.20.0, https://github.com/OpenGene/fastp). Low-quality reads (length < 50 bp or quality score < 20) were removed to retain high-quality sequences. Host DNA contamination was filtered by aligning reads to host sequences using the Bayesian average model (BWA, version 0.7.17, <http://bio-bwa.sourceforge.net).> High-quality reads were assembled using MEGAHIT (version 1.1.2, https://github.com/voutcn/megahit), and contigs ≥ 300 bp were selected as final assembly results. ORF prediction was performed using Prodigal (version 2.6.3, https://github.com/hyattpd/Prodigal), and predicted genes with ≥90% sequence identity were clustered using CD-HIT (version 4.7, http://weizhongli-lab.org/cd-hit/). High-quality reads were mapped to the non-redundant gene set using SOAPaligner (version soap2.21, <https://github.com/ShujiaHuang/SOAPaligner).> Bray-Curtis distance-based Principal Coordinates Analysis (PCoA) was used to evaluate species dispersion. Differential abundance among taxa was analyzed using a combination of LEfSe (Linear Discriminant Analysis Effect Size) and ALDEx2 (Analysis of Differential Abundance taking compositionality into account). For ALDEx2, both effect size and FDR-adjusted p-values were calculated. Taxa with an FDR < 0.1 were considered significantly differentially abundant and were displayed in the corresponding figures.

**Selective culture of *Lysinibacillus***

Mouse fecal samples were cultured aerobically in *Lysinibacillus*-specific medium containing 5 mg/L MnSO_4_·H_2_O at 37℃ with shaking at 220 rpm. Metagenomic sequencing was used to confirm the bacterial presence.

**Treatments of *Lysinibacillus sphaericus* under different conditions**

(1) Supernatant collection: The bacterial culture was centrifuged at 4℃, 4,000×g for 10 minutes, and the supernatant was collected. (2) Heat-Killed *L. sphaericus*: The *L. sphaericus* was incubated at 95℃ for 150 minutes, centrifuged, and resuspended in BHI medium. (3) Heated supernatant: The bacterial supernatant was incubated at 95℃ for 30 minutes. (4) ZnCl_2_ treatment: ZnCl_2_ (20 mg/mL) was added to the bacterial culture. (5) LPA treatment: LPA (20 mg/mL) was added to the bacterial culture. (6) Freeze-thaw cycles: The bacterial suspension was frozen rapidly in liquid nitrogen and thawed at room temperature. This process was repeated at least three times. (7) DNase and RNase treatment: DNase (100 µg/mL) or RNase (150 µg/mL) was added to the bacterial suspension and incubated at 37℃ for 2 hours. (8) BNPP treatment: BNPP (20 mg/mL) was added to the bacterial suspension. (9) Molecular sieve filtration: The bacterial suspension was processed using Amicon® Ultra devices (3,000, 10,000, 30,000, 50,000, and 100,000 MWCO), and both the upper and lower filtrates were collected for experiments.

**Bacterial Strains and Culture Conditions**

*L. sphaericus* was purchased from ATCC, while *Lysinibacillus* *fusiformis* and *Lysinibacillus macroides* were obtained from BeNa Culture Collection. All strains were cultured aerobically at 37℃ in BHI medium.

**Co-culture of bacteria and cells**

Suspensions of *L. sphaericus* were added to Caco-2 at concentrations of 5%, with BHI medium as a control. Aspirin (4 mM) or DMSO was then added, and the cultures were incubated for 24 hours before sampling.

**Biochemical and ELISA assays**

Serum ALT and AST levels were measured using an automated biochemical analyzer (Mindray). Serum BUN and Cr levels were assessed using BUN and Cr assay kits (Nanjing Jiancheng Bioengineering Institute, Jiangsu, China). CES activity in fecal or bacterial supernatants was measured using a CES ELISA kit (Enzyme-linked Immunosorbent Assay, Jiangsu, China) according to the manufacturer’s instructions.

**Construction of *Escherichia coli* Strains Expressing Vector or *est1*/*est2*/*est3*/*est4*/*estB***

Using the pET28a plasmid as an overexpression vector, the genes encoding carboxylesterases from *L. sphaericus* were expressed in *E. coli*. The gene fragments were amplified and cloned into the pET28a plasmid using XhoI and NdeI double digestion, constructing the *est1*/*est2*/*est3*/*est4*/*estB* plasmids. DNA sequencing was performed to confirm the accuracy of the inserted sequences. The verified plasmids were then introduced into *E. coli* BL21 (DE3), while the empty pET28a vector was introduced into *E. coli* BL21 (DE3) as a control. *E. coli* BL21 (DE3) strains overexpressing *est1*/*est2/est3/est4/estB* or carrying the empty vector were cultured overnight at 37℃ with shaking at 220 rpm in LB medium supplemented with kanamycin.

**Quantification of *E. coli* Colonization by CFU Enumeration**

Fecal samples were freshly collected, weighed, and homogenized in sterile phosphate-buffered saline (PBS) at 100 mg/mL using vigorous vortexing with sterile glass beads to ensure complete dispersion. The homogenates were serially diluted 10-fold in PBS, and appropriate dilutions were plated onto BHI agar supplemented with kanamycin (50 μg/mL) to selectively enumerate plasmid-retaining *E. coli*. Plates were incubated aerobically at 37°C for 18 h, after which colonies on plates containing 30–300 CFU were counted. CFU values were normalized to the initial fecal mass and expressed as CFU per gram of feces, with a detection limit of approximately 100 CFU/g under these conditions. PBS process blanks and fecal samples collected from pre-gavage mice served as negative controls.

**Purification of protein**

To obtain the microbial EstB, a recombinant plasmid with a C-terminal 6×His tag was transformed into *E. coli* Rosetta (DE3) competent cells. Protein expression was induced with 0.5 mM IPTG. The protein was purified using Ni^2+^-nitrilotriacetic acid affinity resin (Ni-NTA, Qiagen) in a buffer containing 20 mM Tris-HCl (pH 8.0) and 200 mM NaCl, followed by gel filtration chromatography (Superdex-200 10/300 GL, GE Healthcare). The peak fractions were collected and concentrated for crystallization. Purity was assessed using SDS-PAGE.

**Western blot analysis**

Homogenized small intestine tissues or lysed Caco-2 cells were centrifuged at 4℃, 12,000 × g for 15 minutes, and the supernatant was collected. Protein concentration was determined using a BCA kit, followed by the addition of 5×loading buffer and boiling at 100℃ for 7 minutes. Proteins were separated by SDS-PAGE and transferred to PVDF membranes. Membranes were blocked with 5% milk in TBST for 1 hour, incubated overnight at 4℃ with primary antibodies, and then with HRP-conjugated secondary antibodies (CST, 7074, 1:5000) at room temperature for 1 hour. The primary antibodies used were Occludin (ProteinTech, 27260-1-AP, Rabbit, 1:5000), and ZO-1 (ProteinTech, 21773-1-AP, Rabbit, 1:5000).

**Screening of small molecule inhibitors**

The 3D structure of EstB (AlphaFold ID: AF-A0A2X1BQE9-F1) was downloaded from the AlphaFold database. Protein hydrogenation was performed using the Protein Preparation Wizard module, followed by energy optimization using the OPLS2005 force field with an RMSD of 0.30 Å. Receptor grid files centered on key residues (MET30, HIS194, VAL71, and GLN111) were generated using the Receptor Grid Generation module. The MCE bioactive compound library Plus, containing 556,100 compounds, was energy-optimized using LigPrep (Schrödinger, LLC). Virtual screening was conducted with the Virtual Screening Workflow module. High-throughput screening (HTVS) mode in the Glide module was used for initial screening, selecting the top 5–15% of compounds. Standard precision mode was applied in the second round, followed by extra precision mode in the final round to rank compounds. The top 30 compounds were purchased from MCE for experimental validation.

**Statistical Analysis**

Quantitative analysis of IHC and IF was performed using ImageJ software. Statistical analysis was conducted using Statistical Product and Service Solutions (SPSS) 22.0 (Chicago, IL, USA) and Prism 9 (GraphPad Software, San Diego, CA, USA). Two-tailed unpaired Student’s t-tests or one-way ANOVA were used for parametric data, while the Mann-Whitney *U* test or Kruskal-Wallis test was employed for non-parametric data. Statistical significance is denoted as follows: **p* < 0.05, ***p* < 0.01, ****p* < 0.001, and ns indicates no significant difference.

**References**

1. Kato H, Yoshimoto K, Kobayashi M, et al. Oral administration of ethanol with aspirin increases the concentration of salicylic acid in plasma and organs, especially the brain, in mice. Eur J Pharmacol 2010;635:184-7.
2. Li, T, Ding, N, Guo, H, et al. A gut microbiota-bile acid axis promotes intestinal homeostasis upon aspirin-mediated damage. Cell Host Microbe. 10.1016/j.chom.2023.12.015.
3. Chiu CJ, McArdle AH, Brown R, et al. Intestinal mucosal lesion in low-flow states. I. A morphological, hemodynamic, and metabolic reappraisal. Arch Surg 1970;101:478-83.
   4. Zhang W, Zhou B, Yang X, et al. Exosomal circEZH2_005, an intestinal injury biomarker, alleviates intestinal ischemia/reperfusion injury by mediating Gprc5a signaling. Nat Commun 2023;14:5437.

5. Wang K, Zhang Z, Hang J, et al. Microbial-host-isozyme analyses reveal microbial DPP4 as a potential antidiabetic target. Science 2023;381:eadd5787.

6. Trinder P. Rapid determination of salicylate in biological fluids. Biochem J 1954;57:301-3.**Supplementary figures**

**
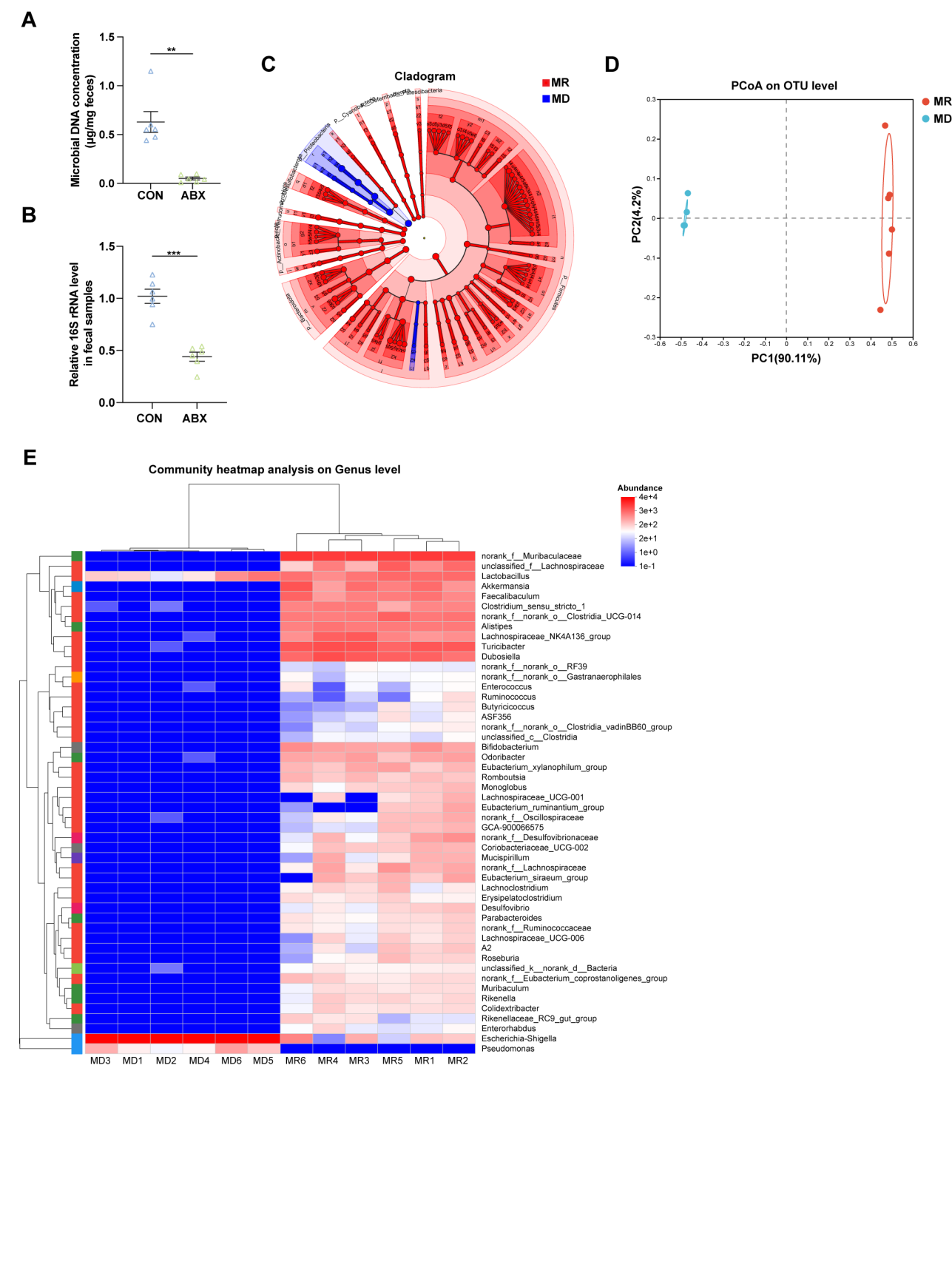
**

**Supplementary fig 1. Validation of antibiotic-mediated gut microbiota depletion and fecal microbiota transplantation efficacy. A** Comparison of fecal DNA concentrations in the CON group versus the ABX group (n=6). **B** Comparison of fecal 16S rRNA relative levels between the CON and ABX groups (n=6). **C** Cladogram representation of fecal microbiota composition in the MR and MD groups (n=6). **D** Principal coordinates analysis (PCoA) of fecal microbiota in the MR and MD groups (n=6). **E** Genus-level community heatmap of fecal microbiota in the MR and MD groups (n=6). ***p* < 0.01. ****p* < 0.001. CON: Control; ABX: Antibiotic.


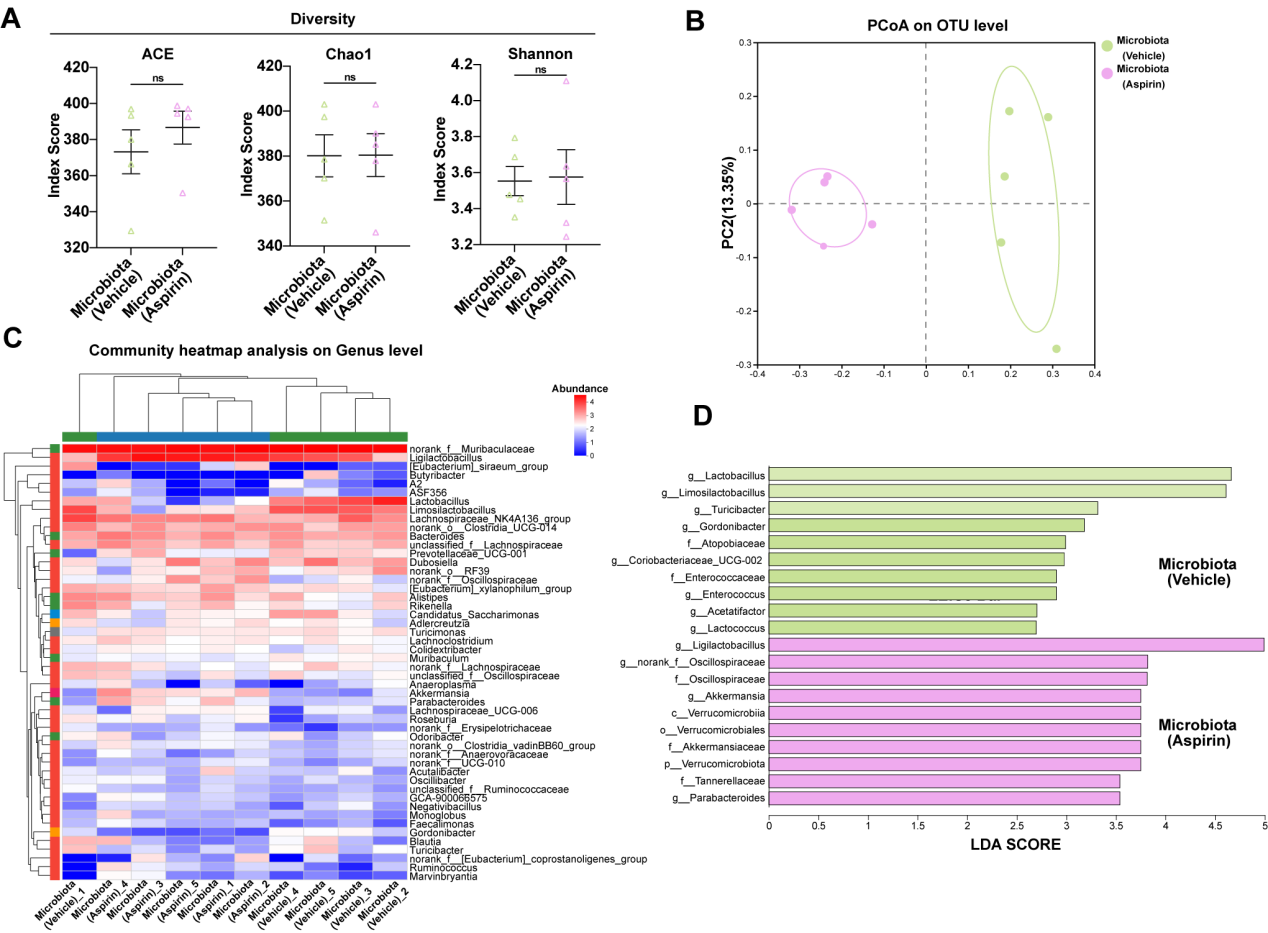


**Supplementary fig 2. 16S rRNA sequencing analysis of gut microbiota alterations induced by aspirin treatment. A** Comparison of diversity in the Microbiota (Vechie) group versus the Microbiota (Aspirin) group based on ACE, Chao1, and Shannon indices (n=5). **B** Comparison of PCoA in the Microbiota (Vechie) group versus the Microbiota (Aspirin) group (n=5). **C** Community heatmap analysis on genus level in the Microbiota (Vechie) group versus the Microbiota (Aspirin) group (n=5). **D** LEfSe analysis revealed distinct bacterial profiles between the two groups (n=5). *p* values were determined by Two-tailed unpaired t-test. ns indicates no significant difference.


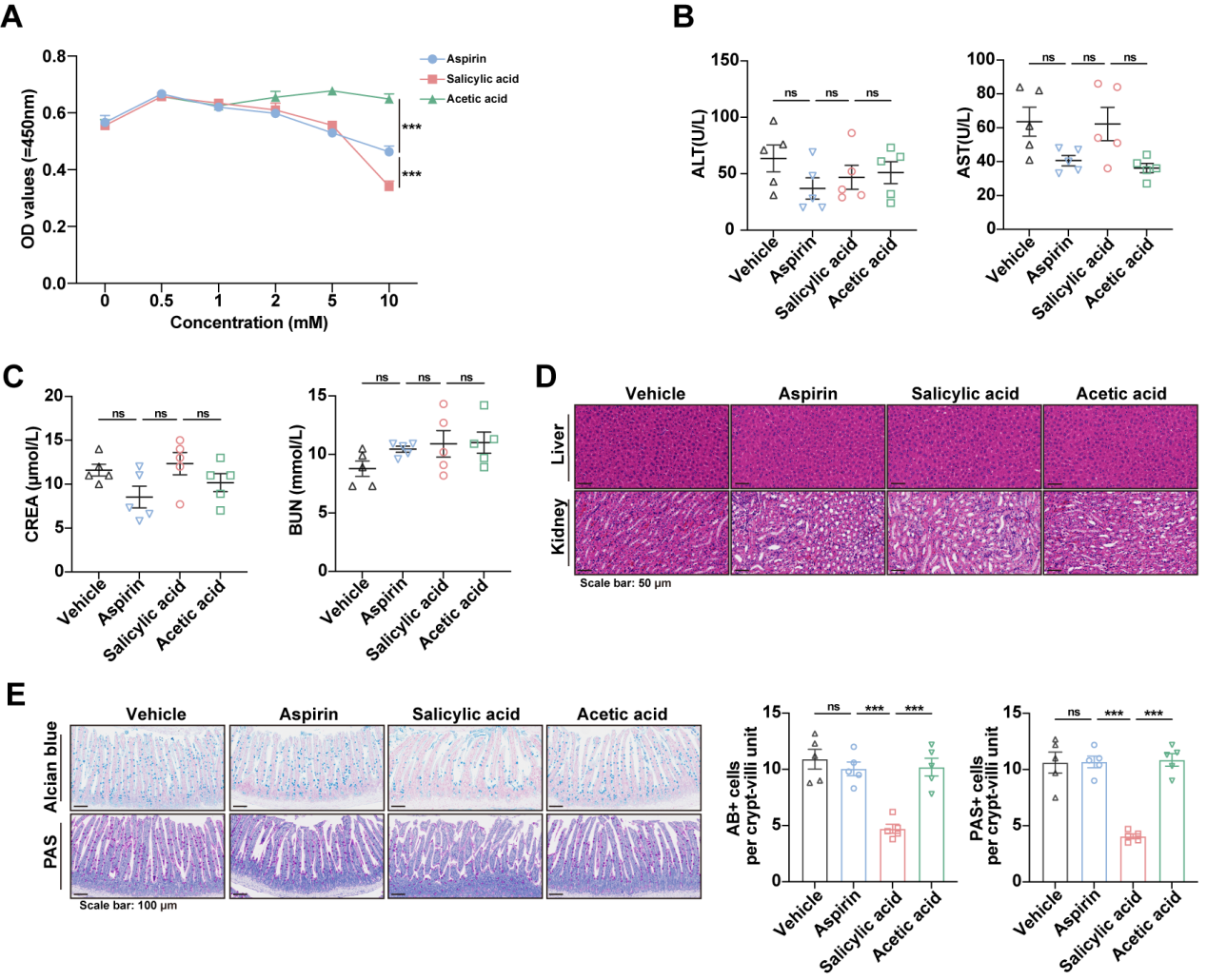


**Supplementary fig 3. Severity of intestinal injury induced by aspirin and its hydrolysates. A** CCK-8 assay was used to detect the effects of different concentrations of DMSO, aspirin, salicylic acid, or acetic acid (0.5-10 mM) on the proliferation of Caco-2 cells (n=3). **B & C** Levels of ALT, AST (B), BUN, and Cr (C) in serum of mice (n=5). **D** H&E staining of liver and kidney tissues(n=5). **E** Alcian blue and PAS images with quantification of AB+ and PAS+ cells per crypt-villus (n=5). *p* values were determined by one-way ANOVA test. ****p* < 0.001; ns indicates no significant difference.


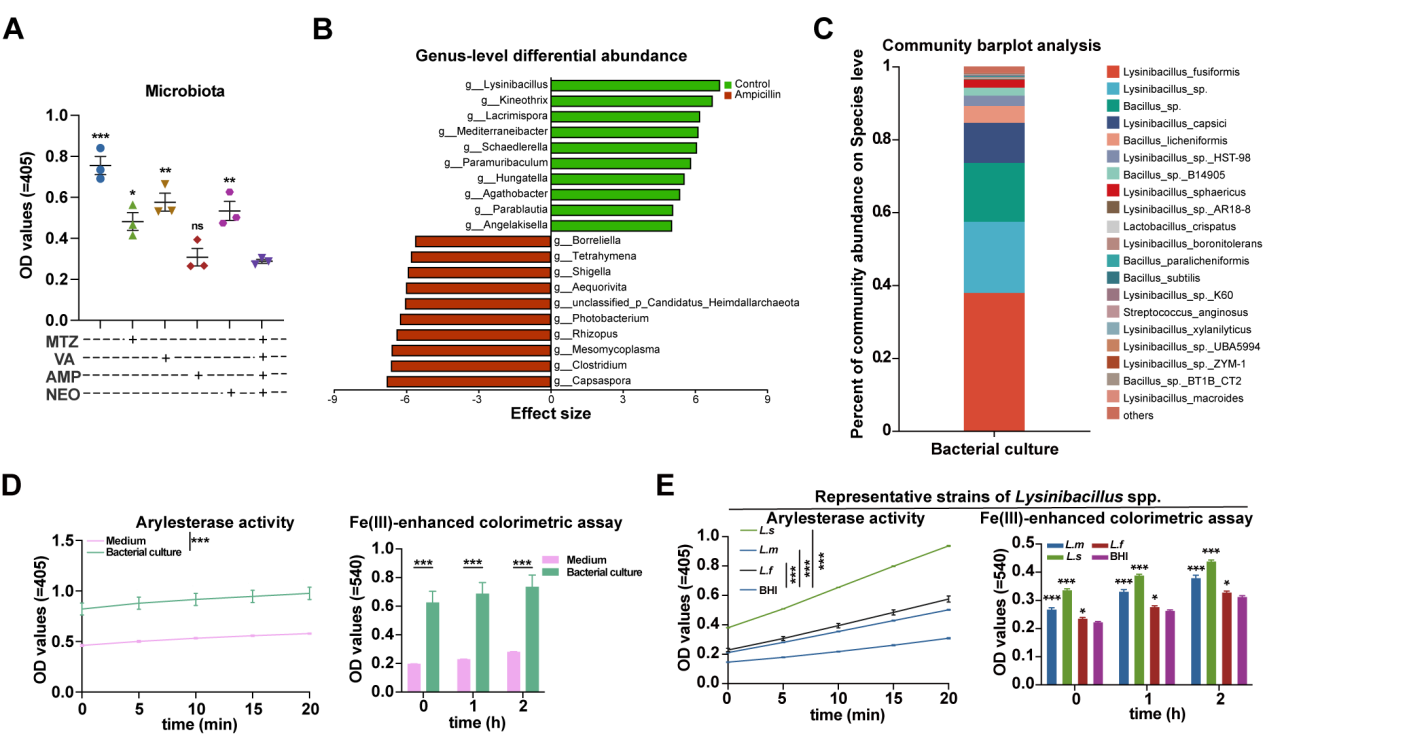


**Supplementary fig 4. Effects of ampicillin on arylesterase activity and gut microbiota composition in mouse feces. A** Effects of in vitro treatment with different antibiotics (MTZ, VA, AMP, NEO) on arylesterase activity in mouse fecal samples (n=3). **B** ALDEx2 analysis of microbial composition at the genus level (n=5). **C** Community bar plot analysis at the genus level of bacterial suspensions cultured in *Lysinibacillus*-specific medium. **D** Arylesterase activity of bacterial suspensions cultured in *Lysinibacillus*-specific medium (n=3) and salicylic acid production after incubation of aspirin with these bacterial suspensions (n=3). **E** Comparison of arylesterase activity among representative *Lysinibacillus* species, including *Lysinibacillus sphaericus* (*L.s*), *Lysinibacillus macroides* (*L.m*), and *Lysinibacillus fusiformis* (*L.f*) (n = 3, Left), and salicylic acid production after incubation of aspirin with these species (n = 3, Right). *p* values were determined by one-way ANOVA test. **p* < 0.05; ***p* < 0.01; ****p* < 0.001. MTZ: Metronidazole; VA: Vancomycin; AMP: Ampicillin; NEO: Neomycin.


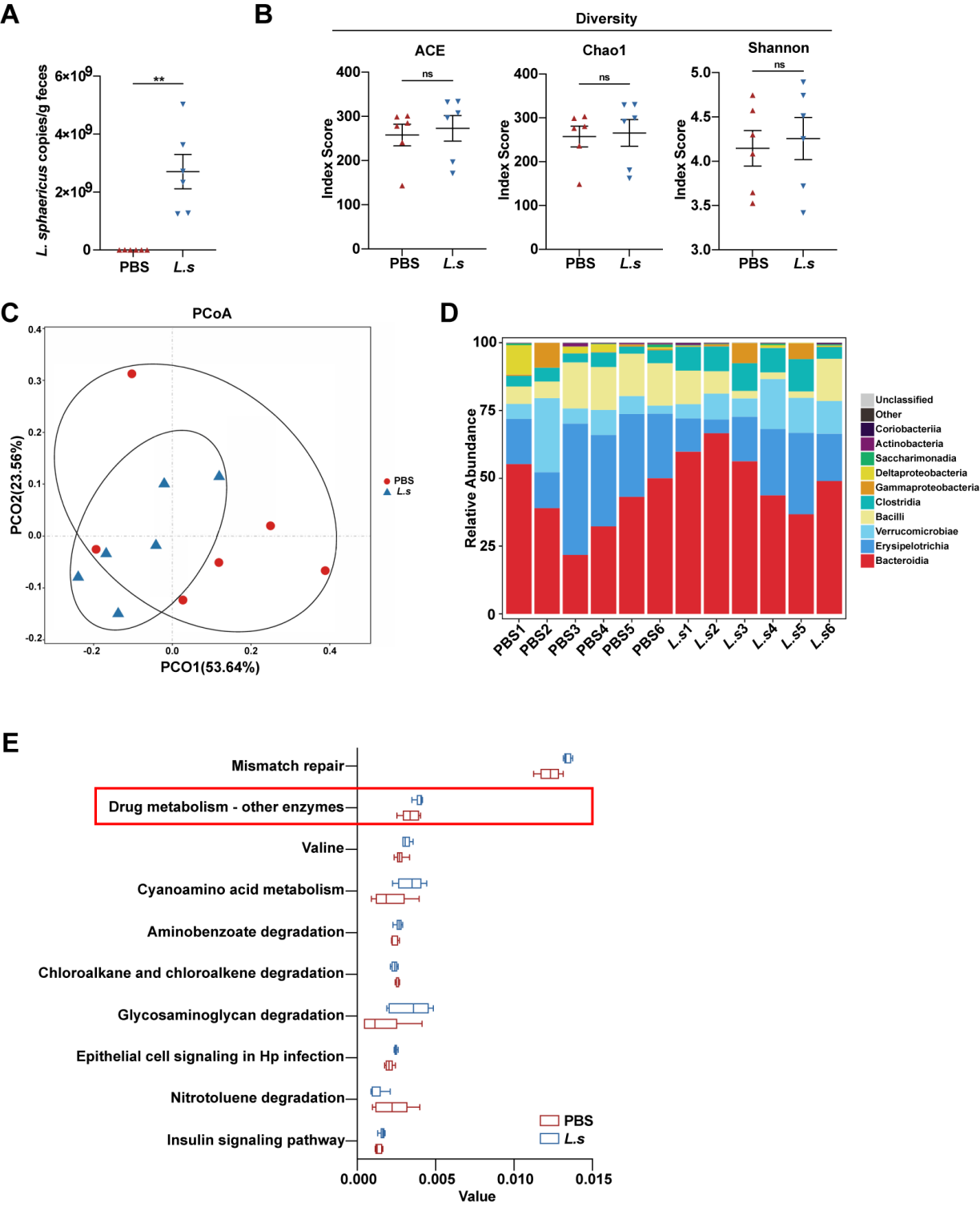


**Supplementary fig 5. 16S rRNA sequencing of feces after gavage with *Lysinibacillus sphaericus* in mice. A** Relative abundance of *L. sphaericus* detected by qPCR. **B** Comparison of diversity in feces of mice gavaged with PBS or *L. sphaericus* based on ACE, Chao1, and Shannon indices (n=6). **C** Comparison of PCoA in the PBS group versus the *L. sphaericus* group (n=6). **D** Community heatmap analysis at the class level in the PBS group versus the *L. sphaericus* group (n=6). **E** Tax4Fun pathway analysis of feces from mice gavaged with PBS or *L. sphaericus* (n=6). *p* values were determined by Two-tailed unpaired t-test. ns indicates no significant difference; ***p* < 0.01. *L.s*: *Lysinibacillus sphaericus*.


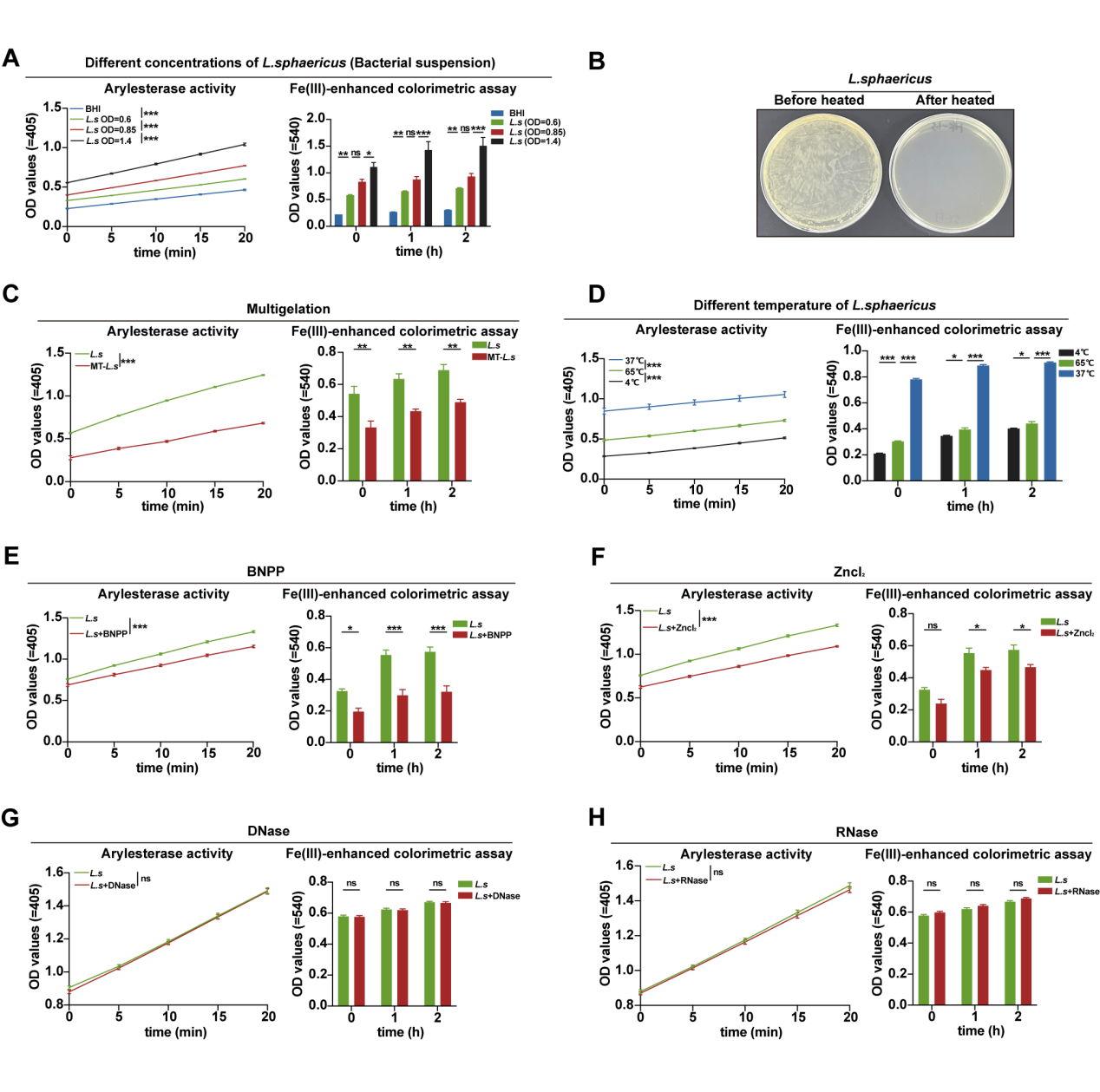


**Supplementary fig 6. Identification of the components in *Lysinibacillus sphaericus* that are responsible for hydrolyzing aspirin. A** Assessment of arylesterase activity in varying concentrations of *L. sphaericus* (Bacterial suspension) and quantification of salicylic acid levels following incubation with aspirin (n=3). **B** Verification of *L. sphaericus* viability after plating heat-killed bacterial suspensions. **C** Assessment of arylesterase activity in *L. sphaericus* following repeated freeze-thaw cycles and quantification of salicylic acid levels after incubation with aspirin (n=3). **D** Detection of arylesterase activity of *L. sphaericus* at different temperatures and detection of salicylic acid content after incubation with aspirin (n=3). **E-H** Analysis of arylesterase activity in *L. sphaericus* and quantification of salicylic acid levels after incubation with aspirin in the presence of BNPP (E), ZnCl_2_ (F), DNase (G), and RNase (H) (n=3). *p* values were determined by two-way ANOVA test. ns indicates no significant difference; **p* < 0.05; ***p* < 0.01; ****p* < 0.001. BNPP: benzyl-2-nitrophenyl phosphate.


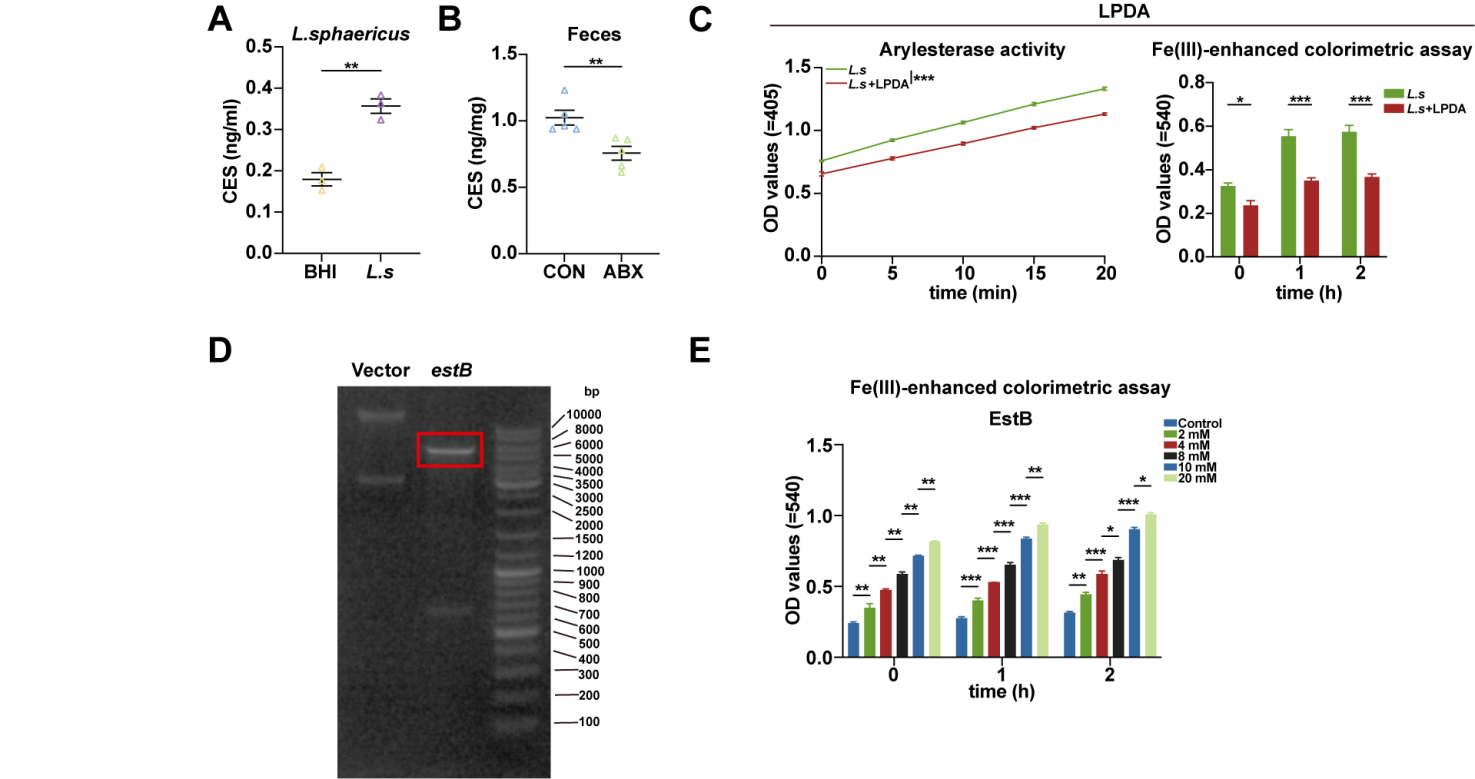


**Supplementary fig 7. EstB-encoded carboxylesterase secreted by *Lysinibacillus sphaericus* mediate aspirin hydrolysis. A & B** Quantification of CES levels in *L. sphaericus* (A) and mouse feces (B) using ELISA (n=3-5). **C** The effect of loperamide on the arylesterase activity of *L. sphaericus* and its ability to hydrolyze aspirin (n=3). **D** PCR validation of the *estB* in *estB-E. coli*. **E** Measurement of salicylic acid levels following incubation of purified EstB with aspirin (n=3). *p* values were determined by Two-tailed unpaired t-test and two-way ANOVA test. **p* < 0.05; ***p* < 0.01; ****p* < 0.001. CES: carboxylesterase; LPDA: loperamide.


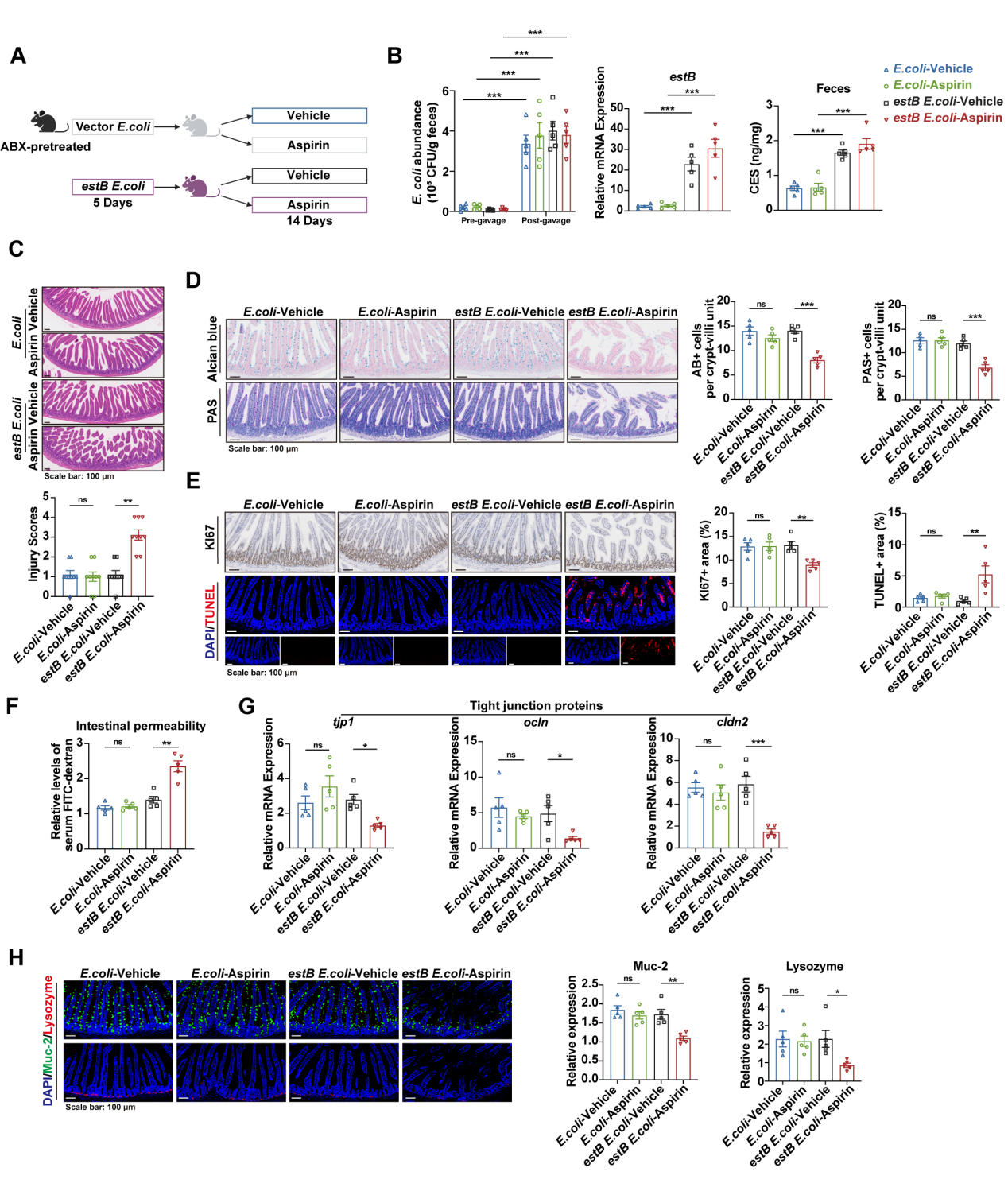


**Supplementary fig 8. *L. sphaericus*-derived EstB deteriorates aspirin-induced intestinal injury. A** Schematic diagram of the mouse experiment involving gavage with vector-*E. coli* or *estB-E. coli* followed by aspirin gavage (Letf). Created with BioRender.com. **B** CFU of plasmid-bearing *E. coli* recovered from feces 48 h after the final gavage on kanamycin-containing BHI plates, compared to pre-gavage controls (left). Relative abundance of the *estB* gene in fecal samples collected 48 h after the final gavage measured by qPCR (middle). Quantification of CES levels in mouse feces collected 48 h after the final gavage by ELISA (right) (n = 5). **C** H&E images and injury scores (n=9) (Right). **D** Alcian blue and PAS images (n=5). **E** Ki67 and TUNEL staining images (n=5). **F** Serum levels of FITC-dextran (n=5). **G** Expression of *tjp1, ocln, and cldn2* in the small intestine (n=5). **H** Immunofluorescence of Muc2 (green) and lysozyme (red), with positive cell counts (n= 5). *p* values were determined by one-way ANOVA and Kruskal-Wallis test. ns indicates no significant difference; **p* < 0.05; ***p* < 0.01; ****p* < 0.001.


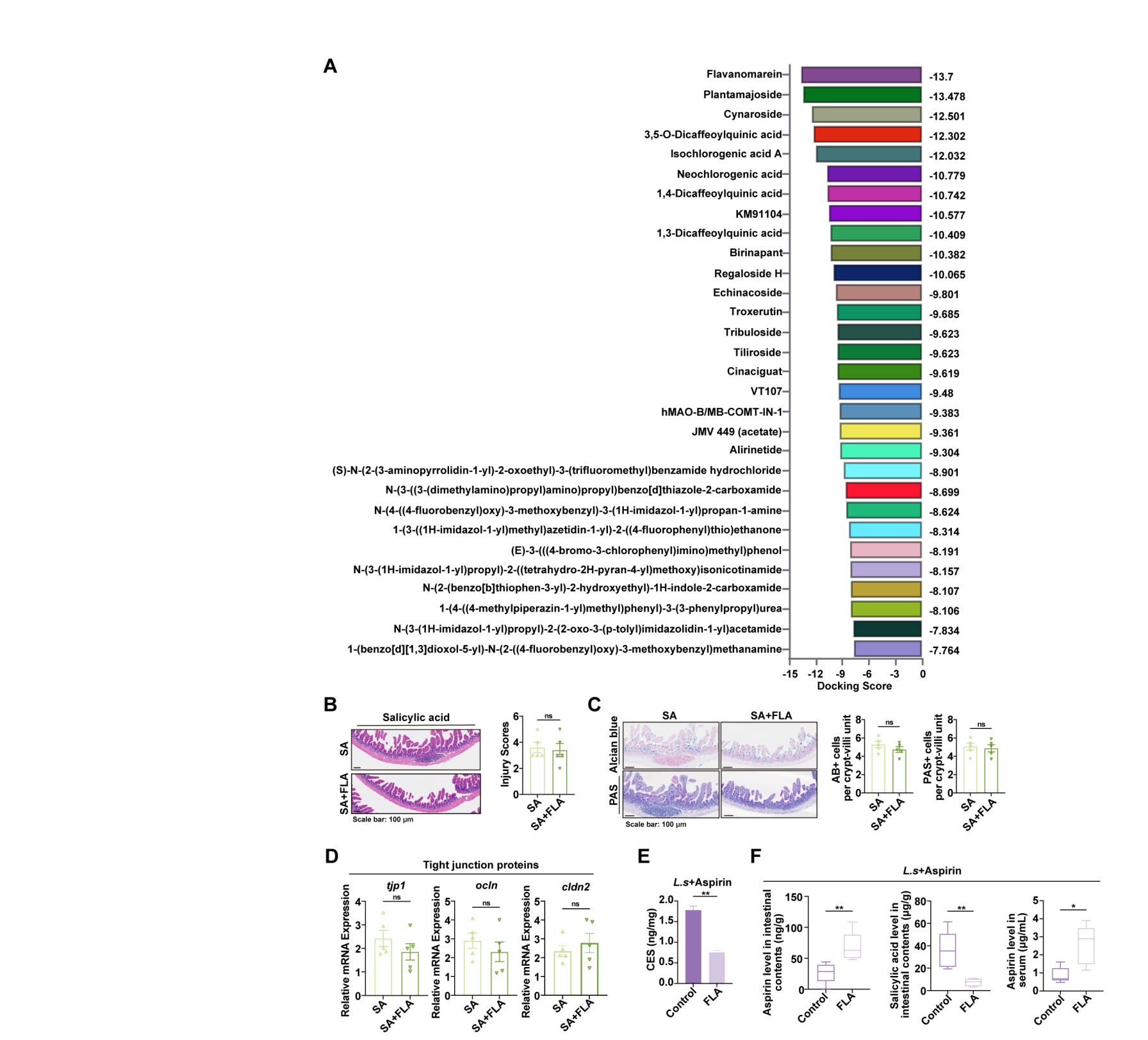


**Supplementary fig 9. FLA screening and its effects on aspirin- or salicylic acid-treated mice A** Top 30 docking hits from virtual screening of 55,610 compounds in the MCE library. **B** Effects of FLA treatment on salicylic acid–treated mouse intestines, shown by H&E staining and corresponding injury scores (n=5). **C** Alcian blue and PAS staining of mouse intestines (n=5). **D** mRNA expression of *tjp1*, *ocln*, and *cldn2* in the small intestine (n=5). **E** Quantification of CES levels using ELISA (n=5). **F** LC-MS detection of aspirin and salicylic acid in intestinal contents, and serum aspirin levels in mice 2 h after oral gavage of aspirin (2 mmol/kg), evaluating the effect of FLA on *L. sphaericus*-mediated aspirin hydrolysis. *p* values were determined using the Mann-Whitney U test or two-tailed unpaired *t*-test. ns indicates no significant difference; **p* < 0.05; ***p* value < 0.01

**Supplemental Table 1. Types of hydrolases.**

| Hydrolyase | Enzyme number |
| --- | --- |
| Dipeptidyl peptidase 4 | EC: 3.4.14.5 |
| Carboxylesterase | EC: 3.1.1.1 |
| Membrane alanyl aminopeptidase | EC: 3.4.11.2 |
| Arylesterase | EC: 3.1.1.2 |
| Carboxypeptidase U | EC: 3.4.17.20 |
| Adenosine deaminase | EC: 3.5.4.4 |
| Gamma-glutamyl hydrolase | EC: 3.4.19.9 |
| Kynureninase | EC: 3.7.1.3 |
| Soluble epoxide hydrolase | EC: 3.3.2.10 |
| Memapsin 2 | EC: 3.4.23.46 |
| Cathepsin B | EC: 3.4.22.1 |
| Triacylglycerol lipase | EC: 3.1.1.3 |
| Acetylcholinesterase | EC: 3.1.1.7 |
| α-Amylase | EC: 3.2.1.1 |
| Alpha-galactosidase | EC: 3.2.1.22 |
| L-asparaginase | EC: 3.5.1.1 |
| Methionine aminopeptidase | EC: 3.4.11.18 |
| Trypsin | EC: 3.4.21.4 |
| Dihydropyrimidinase | EC: 3.5.2.2 |
| Granzyme B | EC: 3.4.21.79 |
| Adenosylhomocysteinase | EC: 3.13.2.1 |
| GTP cyclohydrolase 1 | EC: 3.5.4.16 |
| Hyaluronoglucosaminidase | EC: 3.2.1.35 |
| Caspase-1 | EC: 3.4.22.36 |
| 5'-nucleotidase | EC: 3.1.3.5 |
| Cerebroside-sulfatase | EC: 3.1.6.8 |
| Prolyl oligopeptidase | EC: 3.4.21.26 |
| Arginase | EC: 3.5.3.1 |
| Beta-galactosidase | EC: 3.2.1.23 |
| Guanine deaminase | EC: 3.5.4.3 |
| Inositol-phosphate phosphatase | EC: 3.1.3.25 |
| Alkaline phosphatase | EC: 3.1.3.1 |
| Phospholipase A2 | EC: 3.1.1.4 |
| Beta-glucosidase | EC: 3.2.1.21 |
| Insulysin | EC: 3.4.24.56 |
| L-glutaminase | EC: 3.5.1.2 |
| Alpha-glucosidase | EC: 3.2.1.20 |
| Peptidyl-dipeptidase | EC: 3.4.15.1 |
| Fructose-bisphosphatase | EC: 3.1.3.11 |
| Chitinase | EC: 3.2.1.14 |

**Supplemental Table 2. List of primers used in this study.**

| Primer name | Sequence |
| --- | --- |
| 16S (F) | 5'-ACTCCTACGGGAGGCAGCAGT-3' |
| 16S (R) | 5'-ATTACCGCGGCTGCTGGC-3' |
| Mouse *gapdh* (F) | 5'-TTGATGGCAACAATCTCCAC-3' |
| Mouse *gapdh* (R) | 5'-CGTCCCGTAGACAAAATGGT-3' |
| Mouse *ocln* (F) | 5'-TTGAAAGTCCACCTCCTTACAGA-3' |
| Mouse *ocln* (R) | 5'-CCGGATAAAAAGAGTACGCTGG-3' |
| Mouse *cldn2* (F) | 5'-CAACTGGTGGGCTACATCCTA-3' |
| Mouse *cldn2* (R) | 5'-CCCTTGGAAAAGCCAACCG-3' |
| Mouse *tjp1* (F) | 5'-GCCGCTAAGAGCACAGCAA-3' |
| Mouse *tjp1* (R) | 5'-GCCCTCCTTTTAACACATCAGA-3' |
| *L. sphaericus* (F) | 5'-GTGCAAGATAGCGCAATGGA-3' |
| *L. sphaericus* (R) | 5'-CCGCTAAAGACGCCTTGGAT-3' |
| *estB* (F) | 5'-AGTGTAGCGGTGAAATGCGT-3' |
| *estB* (R) | 5'-TTTCAGTCTGCGACCGTACTC-3' |
